# Strong immune responses and robust protection following a novel protein in adjuvant tuberculosis vaccine candidate

**DOI:** 10.1038/s41598-024-84667-8

**Published:** 2025-01-13

**Authors:** Marcellus Korompis, Christopher J De Voss, Shuailin Li, Alexandre Richard, Salem Salman Almujri, Alberta Ateere, Géraldine Frank, Céline Lemoine, Helen McShane, Elena Stylianou

**Affiliations:** 1 The Jenner Institute, University of Oxford, Oxford, UK; 2 Vaccine Formulation Institute, Rue du Champ-Blanchod 4, 1228 Plan-les-Ouates, Switzerland; 3 Department of Pharmacology, College of Pharmacy, King Khalid University, 61421, Asir-Abha, Saudi Arabia

**Keywords:** Tuberculosis, Vaccine, Heterologous, Viral vector, Adjuvants, Protection, Inactivated vaccines, Protein vaccines

## Abstract

BCG remains the only licensed vaccine for tuberculosis (TB), but its efficacy wanes over time. Subunit vaccines, aim to improve BCG immunity and protection, by inducing responses to a few mycobacterial antigens delivered with a specific platform. Since the platform shapes the immune response induced, selecting the right platform has been challenging due to the lack of immune correlates of protection. Recently, the protein-adjuvated subunit vaccine. M72/AS01E, demonstrated 49.7% efficacy in preventing active TB in latently infected adults, indicating that protective immunity through subunit vaccines is possible. In this study we evaluated the immunogenicity and efficacy of the promising mycobacterial antigen PPE15, formulated with five adjuvants developed by the Vaccine Formulation Institute. While all adjuvants were immunogenic, PPE15 with LMQ protected vaccinated mice against an in vivo *Mycobacterium tuberculosis* challenge, both as a standalone vaccine and as a boost to BCG. Vaccinated mice had enriched lung parenchymal antigen-specific CD4 + CXCR3 + KLRG1− T cells previously associated with TB protection. Heterologous vaccination strategies were also explored by combining intranasal ChAdOx1.PPE15 viral vector, with intramuscular PPE15-LMQ resulting in improved protection compared to individual vaccines. These findings support the progression of this vaccine candidate to the next stages of development.

**Supplementary Information:**

The online version contains supplementary material available at 10.1038/s41598-024-84667-8.

## Introduction

Tuberculosis (TB), caused by *Mycobacterium tuberculosis* (*M. tb*) is a significant public health challenge as a leading cause of morbidity and mortality worldwide. In 2022, an estimated 10.6 million people developed TB globally^1^, highlighting the pervasive nature of this disease. The *Bacillus Calmette-Guérin* (BCG) vaccine, developed nearly a century ago, is the only licensed vaccine available for TB prevention^2^. While BCG has demonstrated efficacy in preventing severe forms of TB in children, its effectiveness wanes over time, particularly against pulmonary TB, which constitutes the majority of TB cases in adults^1,3,4^. This limitation underscores the urgent need for new TB vaccines that will boost the protective efficacy of BCG, especially in regions with high TB prevalence^5^. Adding to the complexity of TB control efforts, *M. tb*, and in particular rifampicin-resistant strains, have been assigned as a critical group on the WHO Bacterial Priority Pathogen List 2024^6^, highlighting the even greater need for an effective vaccine. Recent advancements in TB vaccine research have shown promising results with an adjuvanted protein vaccine, M72/AS01E, which conferred 49.7% protection against active TB disease in latently infected adults^7^. 

PPE15 (Rv1039c) is a mycobacterial protein that is part of the *M. tb* ESX-5a secretion system, which is essential for bacterial homeostasis and entry into a dormant state^8–10^. Recognised by CD8 + and CD4 + T cells in individuals with active and latent TB infections, PPE15 has emerged as a promising antigen for eliciting protective immune responses^11,12^. Previous studies have demonstrated the strong potential of PPE15, when delivered intranasally via a replication-deficient chimpanzee adenovirus vector (ChAdOx1), to confer protection against *M. tb* infection, either alone or as a boost to BCG vaccination in mice^13,14^. Building on this progress, our study focuses on delivering PPE15 as part of an adjuvanted formulation. Considering the need for adjuvants to be openly accessible, we have established a collaboration with the Vaccine Formulation Institute (VFI). VFI develops clinically relevant adjuvants and ensures their availability to the vaccine community through an open-access model, promoting the development and equitable access of adjuvanted vaccines, especially in low- and middle-income countries (LMICs) disproportionally burdened by TB and other diseases^15^.

This study investigated the immunogenicity and protective efficacy of PPE15 when formulated with various VFI adjuvants including three squalene oil-in-water emulsion based adjuvants: Sepivac SWE™^16^, SQ^17^ and SMQ^18,19^, and two liposome-based adjuvants: LQ^20^ and LMQ^21^. We evaluated the different formulations for their ability to generate immune responses associated with protection^22,23^ and for efficacy using both the Mycobacterial Growth Inhibition Assay (MGIA)^24^ and in vivo *M. tb* challenge studies in C57BL/6 mice. Additionally, we investigated heterologous prime-boost vaccination strategies using intranasal ChAdOx1.PPE15 to explore potential synergistic protective effects between the two platforms.

Our findings indicate that PPE15-LMQ was immunogenic and protective in both homologous and heterologous regimens and support the progression of this candidate to the next stages of vaccine development.

## Methods

### Mice and immunisations

Six- to eight-week-old female C57BL/6 mice were purchased from Envigo, UK. Animals were group housed in IVCs under SPF conditions, with constant temperature and humidity. Mice were vaccinated with BCG-Pasteur at 3.5 × 10^5^ Colony Forming Unit (CFU) intradermally (i.d.) in the ears of mice (25 µl on each side). BCG was prepared in house in 7H9 broth (Becton Dickinson, UK) supplemented with 10% Middlebrook albumin dextrose catalase and 0.05% polysorbate 80 (Becton Dickinson, UK). Vaccinations with ChAdOx1 constructs were performed intranasally (i.n.) drop-by drop over two nostrils, with 1 × 10^8^ infectious units (ifu) in a 50 µL volume, diluted in endotoxin-free PBS (Merck Life Science, UK). For BCG-boosting experiments, 10 weeks were allowed between BCG-prime and booster vaccination. PPE15 protein was prepared by Biologics Corp, USA and formulated in-house at 1:1 volume ratio with the adjuvants. The adjuvanted formulations were administered intramuscularly as 2 × 50 µl in each hind limb. All procedures were performed in accordance with the UK Animals (Scientific Procedures) Act 1986, under project license number P9804B4F1, granted by the UK Home Office. Animal studies were approved by the Animal Welfare and Ethical Review Board (AWERB), University of Oxford; studies were in accordance with the Animal Research: Reporting of in vivo Experiments (ARRIVE) guidelines. All vaccinations were done under short-term inhalational anaesthesia using vaporised IsoFlo^®^. All animals were humanely sacrificed at the end of each experiment by cervical dislocation.

### Adjuvants and formulations

Sepivac SWE™ was provided by Seppic (France). The SQ, SMQ, LQ and LMQ adjuvant formulations were manufactured at the Vaccine Formulation Institute, Switzerland as described previously^18^. Briefly, SQ adjuvant was prepared by combining squalene-in-water emulsion containing cholesterol (Merck-Sigma, USA) with QS-21 in solution (Desert King International, USA). SMQ was similarly prepared, resulting in a squalene-in-water emulsion containing both cholesterol and synthetic TLR4 ligand 3D-6-acyl-PHAD (3D6AP) (Merck-Avanti, USA). The liposome-based LQ adjuvant was prepared by combining liposomes, made of 1,2-dioleoyl-sn-glycero-3-phosphocholine (DOPC) (Merck-Avanti, USA) and cholesterol, with QS-21 in solution. LMQ adjuvant was prepared similarly to LQ but with 3D6AP included during the liposome preparation. Compatibility between the PPE15 antigen and the adjuvants was confirmed and documented by performing formulation compatibility studies with all adjuvants prior to immunisations. Briefly, the PPE15 antigen was formulated with each adjuvant and the formulations were characterized after 24 h of storage at 5 °C. Physicochemical characteristics of the adjuvants were determined as follows. pH was measured using a pH-meter (Mettler Toledo). Adjuvants particle size and polydispersity were measured by Dynamic Light Scattering (Zetasizer Ultra, Malvern Panalytical). Zeta potential was measured by Electrophoretic Light Scattering (Zetasizer Ultra, Malvern Panalytical). Quantification of the adjuvant components, including DOPC, cholesterol, squalene, 3D6AP, and QS-21, was carried out by liquid chromatography using HPLC-UV (Agilent) and UPLC-MS (Waters) instruments. Integrity of the antigen was assessed through ELISA by coating maxisorp 96-well plates (ThermoFisher Scientific) with 50 µg/ml of PPE15 protein in PBS and SWE, SQ, SMQ, LQ, or LMQ in 1:1 volume ratio. Formulations were incubated overnight at 4 °C, and adjuvant only and PBS only groups, without PPE15, were included as controls. Plates were washed with 0.05% Tween20 (Sigma-Aldrich) in PBS and blocked with 2.5% BSA/PBS for 2 h, followed by a 2-hour incubation with 1/1000 diluted polyclonal mouse serum samples or 1/1000 anti-His antibody (Sigma-Aldrich) prepared in PBS/Tween (PBS/T). Plates were washed and incubated with alkaline phosphatase-conjugated anti-mice IgG (1/5000) (Sigma-Aldrich) diluted in PBS/T for one hour at room temperature. Development was performed with 1 mg/mL of 4-nitrophenylphosphate tablet (Sigma-Aldrich) diluted in diethanolamine buffer (ThermoFisher Scientific). Optical density (OD) was measured at 405 nm (Gen5 software) using a spectrophotometer (BioTeK Microplate Reader).

The final adjuvant and antigen formulations were prepared using PBS (Sigma-Aldrich, Dorset, UK) and combined on the day of administration. Each injectable dose of LQ, SQ, LMQ and SMQ contained 5 µg (50 µg/mL) of QS21 saponin and/or 2 µg (20 µg/mL) of the TRL4 agonist 3D6AP. For the oil-in-water emulsions the adjuvants contained a squalene dose of 1 mg/dose (10 mg/mL) (SWE) and 0.5 mg/dose (5 mg/mL) (SQ/SMQ).

### Immunogenicity

Bronchoalveolar lavage (BAL), lung, and spleen cells were extracted from naïve and vaccinated mice. Lungs were perfused with PBS, chopped into small pieces, and digested with DNase/Collagenase (Sigma) as described previously^25^.

Flow cytometry: Spleen and lung cells were stimulated with media alone or with 2 µg/ml of PPE15 peptide pool, composed of 15-mer peptides overlapping by 11 amino acids, spanning the whole antigen sequence (Peptide Protein Research, UK), before addition of Golgi plug (BD Biosciences) for a further 4 h at 37 °C. Plates were placed at 4 °C overnight and cells stained the following day. Cells were stained with live/dead fixable stain (ThermoFischer Scientific) for 10 min, followed by α-CD16/32 α-CD45R, α-TCRβ, α-CD8 (eBioscience, UK), α-CD4 (Biolegend, UK). Cells were fixed and permeabilised with Cytofix/Cytoperm then stained intracellularly with α- IFN-γ, -TNF-α, -IL-2, -IL-17 (eBioscience, UK) (Supplementary Fig. 1). For tetramer staining, 2 × 10^6^ cells were stained with I-A(b) PPE15_1–15_ phycoerythrin (NIH Tetramer Facility, Atlanta, GA, USA) or H-2D^b^ PPE15_192–200_ -allophycocyanin (ProImmune, Oxford, UK) at 4 °C for 30 min, washed with PBS and stained with α- KLRG1, -CXCR3, -CX3CR1 (BioLegend, UK), and -PD1 (eBioscience, UK) (Supplementary Figs. 2 and 3). Samples were run on a LSR II or LSR Fortessa X-20 flow cytometer, and the data were analysed using FlowJo (TreeStar, Ashland, OR, USA).

Intravascular staining: Mice were injected via the lateral tail vein with 100 µl of α-CD45.2 fluorescein isothiocyanate (eBioscience, UK) at 25 µg/ml. Two minutes later, the lungs were collected and chopped into small pieces using sterile scissors or with C-tubes containing digestive medium (RPMI-1640, SIGMA, with collagenase and DNase). C-tubes were processed using a GentleMACS tissue dissociator (Miltenyi Biotec, UK). The lung tissues were digested for 1 h at 37 °C, after which they were forced through a 100 μm-mesh-size filter (Greiner Bio-One). The cells were washed, red blood cells lysed and filtered using a 40 μm cell strainer (Greiner Bio-One) before final wash and resuspension.

Enzyme-linked immunosorbent assay (ELISA) was used to measure PPE15-specific IgG, IgG1, and IgG2c responses in the serum and IgA in the BAL. Maxisorp 96-well plates (ThermoFisher Scientific) were coated with 2 µg/ml (or 10 µg/ml for BAL IgA) of PPE15 protein in PBS and incubated overnight at 4 °C. Plates were washed with 0.05% Tween20 (Sigma-Aldrich) in PBS and blocked with 2.5% BSA/PBS for 2 h, followed by a 2-hour incubation with serially diluted serum samples prepared in PBS/Tween (PBS/T). Plates were washed and incubated with either alkaline phosphatase-conjugated anti-IgG (1/5000) or anti-IgA (1/1000) (Sigma-Aldrich) or anti-IgG1 (1/1000) or anti-IgG2c (1/1000) (BioRad, CA, USA) diluted in PBS/T for one hour at room temperature. Development was performed with 1 mg/mL of 4-nitrophenylphosphate tablet (Sigma-Aldrich) diluted in diethanolamine buffer (ThermoFisher Scientific). Optical density (OD) was measured at 405 nm (Gen5 software) using a spectrophotometer (BioTeK Microplate Reader).

Mycobacterial growth inhibition assay (MGIA). 5 × 10^6^ spleen cells were incubated with 1000 CFU of BCG Pasteur and RPMI-1640 HEPES modification containing 2 mM L-Glutamine and 10% FBS (Sigma-Aldrich) in a total volume of 600 µl per well in a 48-well plate. Cultures were incubated at 37 °C for 96 h. Extracellular and lysed intracellular BCG was transferred to BACTEC MGIT tubes supplemented with BBL MGIT OADC and PANTA (BD Bioscience, Oxford, UK). Tubes were placed on the BACTEC 960 machine (BD Bioscience, Oxford, UK) and incubated at 37 °C until the detection of positivity by fluorescence. Standard curves (Supplementary Fig. 4) were used convert time to positivity (TTP) to colony forming units (CFU). To confirm inoculum dose, control tubes were set up by inoculating supplemented BACTEC MGIT tubes with the same volume of BCG as the samples on day 0.

### Mycobacterial challenge

A Biaera Aero-MP-controlled nebuliser (Biaera Technologies, USA) was used to infect mice with aerosolised *M. tb* Erdman K01 (TMC107, BEI Resources, USA) at an airflow rate of 12 L/min and a pressure of 20 lb/in^2^ gauge. Infection dose was confirmed 1-hour post-infection using two animals per run (7–38 CFU range).

Lungs and spleen were collected 4 weeks after infection, homogenised in re-inforced tubes (Stretton Scientific) and a Precellys 24 homogeniser. Homogenates were serially diluted in PBS, plated on Modified 7H11 plates (Animal and Plant Health Agency, UK) and incubated at 37 °C for 4 weeks.

### Statistical analysis

All graph and statistical analyses were performed using GraphPad Prism 10 software (GraphPad Software Inc.). Normality data was determined by Shapiro–Wilk test. To determine statistical significance, Mann–Whitney test was used to compare two groups and one-way analysis of variance (ANOVA; Kruskal–Wallis) followed by post hoc Dunnett’s or Dunn’s multiple comparison test for multiple groups. Values of *p* ≤ 0.05 were considered statistically significant. *p ≤ 0.05, **p ≤ 0.01, ***p ≤ 0.001, ****p ≤ 0.0001.

## Results

### Strong antigen-specific Th1-immune responses induced by PPE15 formulated with saponin-based adjuvants

**Fig. 1:**
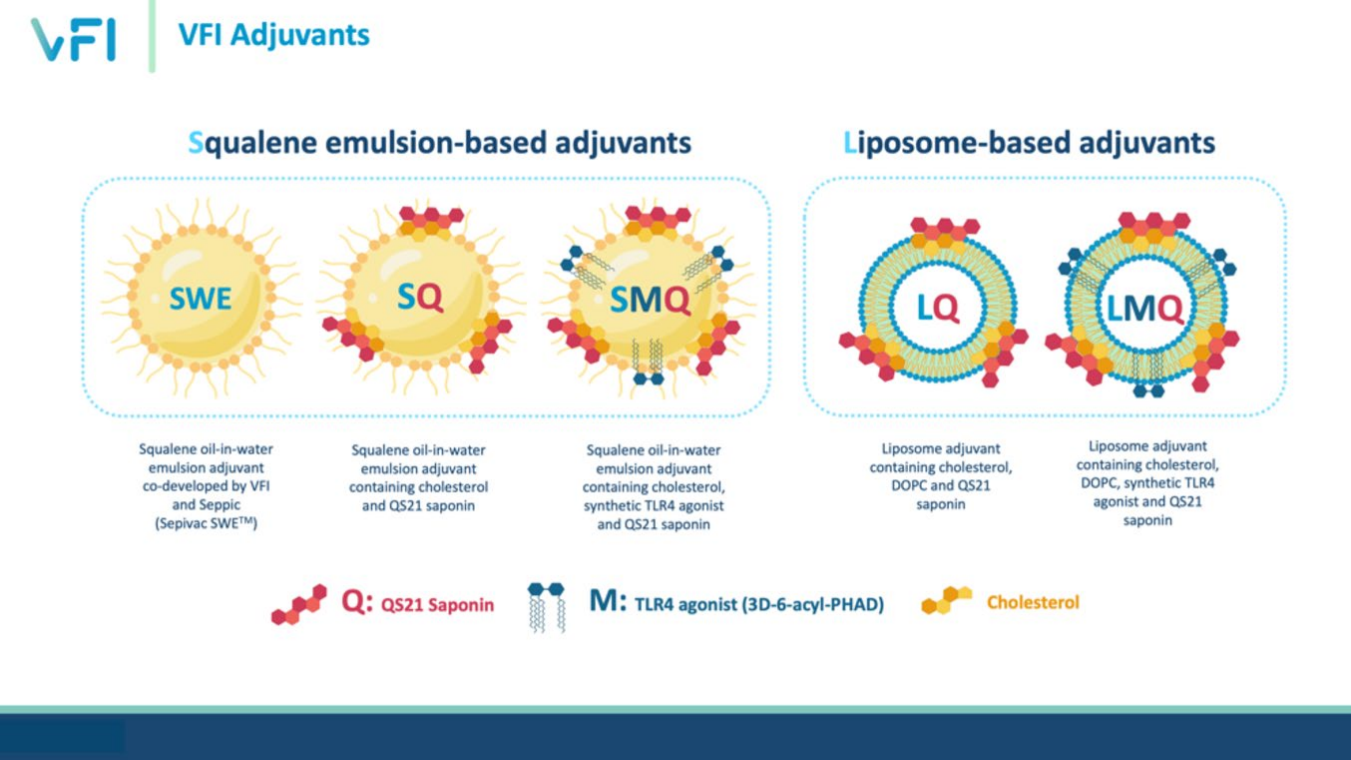
VFI adjuvants composition.

Previous work demonstrated the protective efficacy of PPE15 when delivered as ChAdOx1.PPE15 in mice^13^. In the current study, PPE15 was delivered as a recombinant protein formulated with five adjuvants (SWE, SQ, SMQ, LQ and LMQ) (Fig. 1). To determine their immunogenicity, mice were immunised twice intramuscularly with a 2-week interval. Spleens and sera were collected at the end of the study to measure cellular and antibody responses, respectively.

**Fig. 2:**
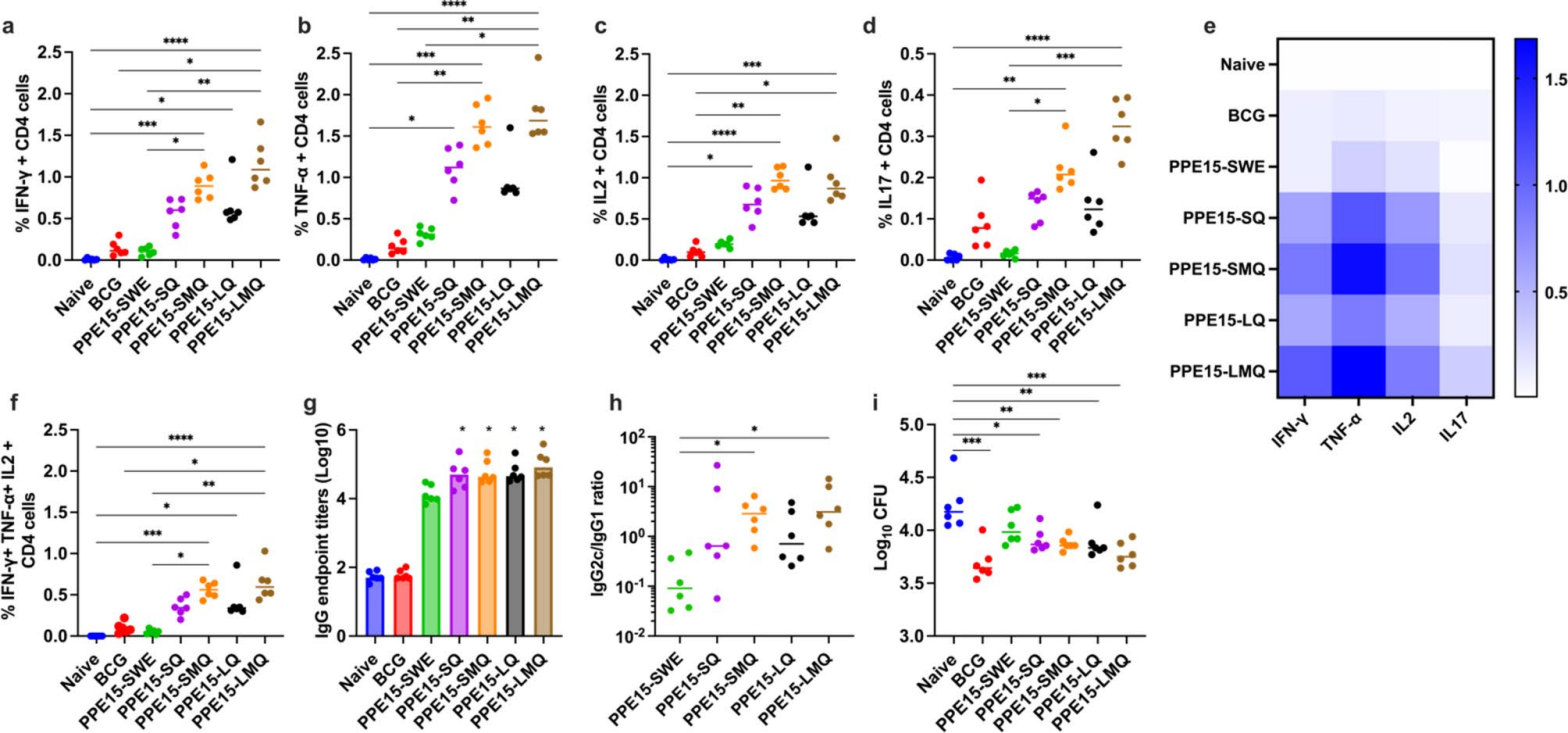
Cellular, humoral responses and in vitro mycobacterial control inhibition following vaccination with adjuvanted PPE15 protein. C57BL/6 mice were vaccinated twice with PPE15 protein formulated in five distinct adjuvants. Two weeks after the last vaccination, splenocytes from vaccinated and control animals were stimulated with PPE15 peptide pool to assess cytokine production. Proportion of CD4 + T cells expressing: (**a**) IFN-γ, (**b**) TNF-α, (**c**) IL-2, (**d**) IL-17. (**e**) A heatmap illustrating the proportion of CD4 + T cells producing each cytokine, bars represent the median response in each group. (**f**) Proportion of CD4 + T cells expressing all three of IFN-γ TNF-α and IL-2. (**g**) Serum PPE15-specific total IgG and (**h**) IgG2c to IgG1 end titre ratio (starting dilution 1:100). Statistical significance compared to the naïve (*). (**i**) MGIA was set up using splenocytes from vaccinated and control animals. Each symbol in the graphs represents an individual animal, *n* = 6 per group. Experiment completed once. The lines indicate median value for each group. Statistical analyses were performed using Kruskal-Wallis, followed by Dunn’s multi-comparison test to evaluate differences between groups. *p < 0.05, **p < 0.01, ***p < 0.001, ****p < 0.0001.

Antigen-specific CD4 + responses were detectable across all vaccinated groups, with a trend for strongest responses in the PPE15-LMQ group (Fig. 2a–e). PPE15-SMQ and LMQ induced a significant percentage of CD4 + T cells expressing IFN-γ, TNF-α, IL-2, and IL-17 compared to the naïve group. LQ induced CD4 + IFN-γ and SQ induced CD4 + TNF-α and IL-2 that was significantly higher compared to the naïve control group. PPE15-specific CD8 + T cell responses were low across all groups. (Supplementary Fig. 5). We next performed a multifunctional cytokine analysis to compare the number of cells secreting all three cytokines: IFN-γ, TNF-α, and IL-2. PPE15-SMQ, -LMQ and -LQ induced a high frequency of triple-positive CD4 + T cells (Fig. 2f).

Since antibodies might also have an important role in protective immunity^26^, we then measured the level of PPE15-specific IgG in the serum of vaccinated mice. A robust antibody response was detected in all groups, with PPE15-SWE, -SQ, -SMQ, -LQ, and -LMQ comparable to one another and significantly higher than in unvaccinated mice, except for PPE15-SWE (Fig. 2g). The IgG2c/IgG1 ratio was greater than 1 in the PPE15-LMQ and PPE15-SMQ, and in both cases was significantly higher than the ratio observed for PPE15-SWE (Fig. 2h).

There has been an increase in the utilisation of mycobacterial growth inhibition assays (MGIA) to down-select vaccine candidates^24^. In the absence of validated immune correlates of protection for TB vaccines, such a strategy not only accelerates vaccine development but also reduces the number of animals to be infected with virulent *M. tb*, in line with the 3Rs principles of replacement, reduction and refinement of animal use in research^27^. We established an MGIA using splenocytes from the same animals to select two adjuvants for further in vivo efficacy testing. We detected a significantly lower CFU using splenocytes from i.d. BCG-vaccinated compared to the naïve group, as well as when using splenocytes from the PPE15-SQ, -SMQ, -LQ and -LMQ (Fig. 2i). These results indicate an in vitro protective signal for all groups with the strongest signal in PPE15-LMQ.

### PPE15-LMQ and -SQ formulations protective against *M. tb* challenge

**Fig. 3:**
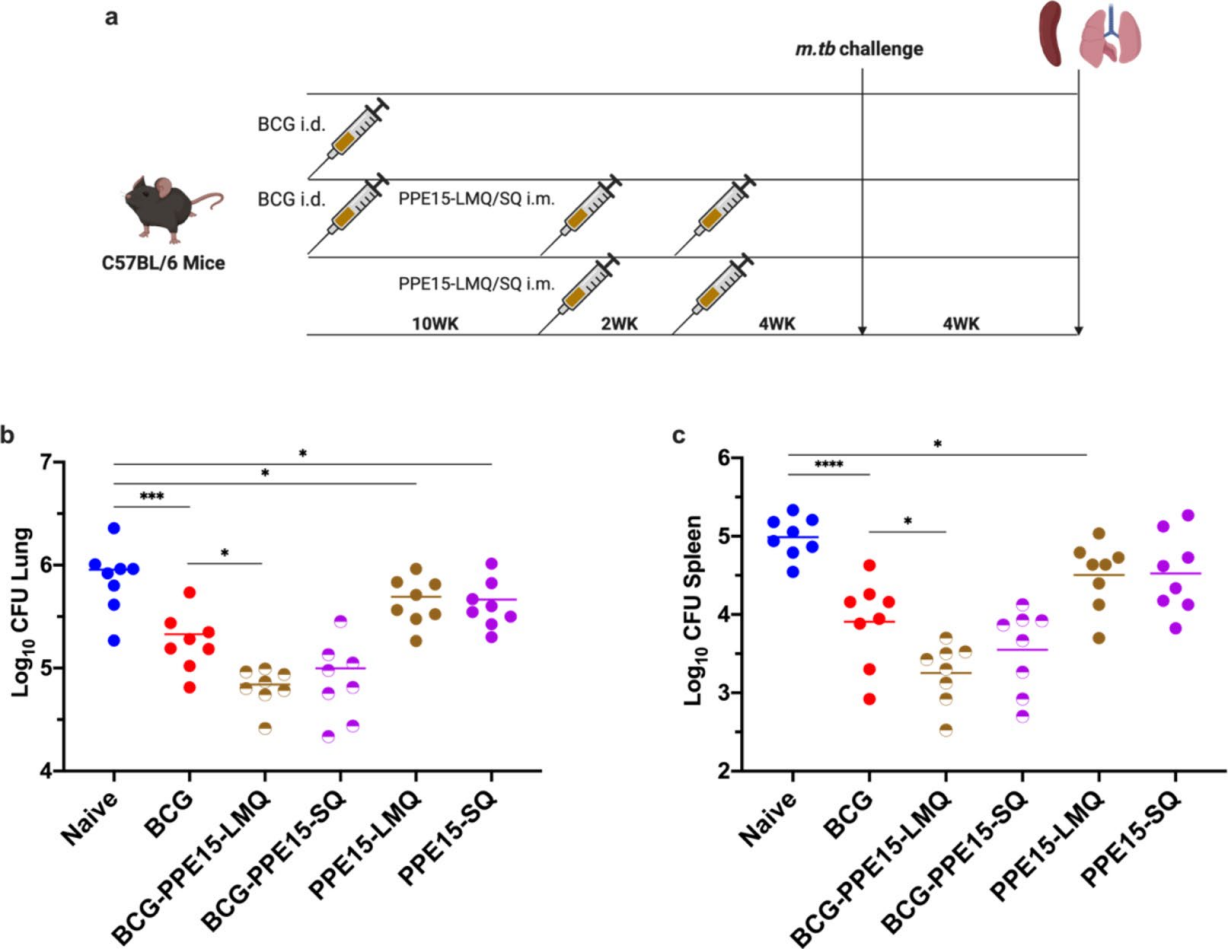
Protective efficacy of PPE15 protein-adjuvanted vaccine candidates. (**a**) Experimental schema (created with BioRender.com). (**b**) Lungs and (**c**) spleens were harvested at the end of the study for colony-forming unit (CFU) enumeration. Each dot represents one animal, *n* = 8 per group. The lines indicate the mean CFU count for each group. Statistical significance was assessed using One-Way ANOVA followed by Dunnett’s multi-comparison test, with significance levels indicated as follows: *p < 0.05, ***p < 0.001, ****p < 0.0001. *i.d.* intradermal, *i.m.* intramuscular. Data are representative of one of two independent experiments.

SQ and LMQ were selected for further in vivo efficacy testing. Despite promising results of SMQ, SQ was selected over SMQ due to its more advanced development for GMP manufacture. Groups of mice were either primed with BCG or left unprimed, and then given booster doses of protein-adjuvant (BCG-PPE15-LMQ/PPE15-LMQ or BCG-PPE15-SQ/PPE15-SQ). Unvaccinated mice or mice receiving only BCG were included as controls. Following immunisations, mice were exposed to aerosolised *M. tb*, and lung and spleen tissues were collected at the end of the study for bacterial enumeration (Fig. 3a).

BCG vaccination provided significant protection in both the lung and spleen compared to the unvaccinated naïve group (Fig. 3b, c). In the lung and spleen, boosting BCG with PPE15-LMQ significantly improved efficacy of BCG. Additionally, standalone vaccinations with PPE15-LMQ or PPE15-SQ, without a BCG prime, also reduced the bacterial load compared to unvaccinated mice (Fig. 3b, c).

### Improved PPE15 responses in the BCG-primed PPE15-LMQ group

**Fig. 4:**
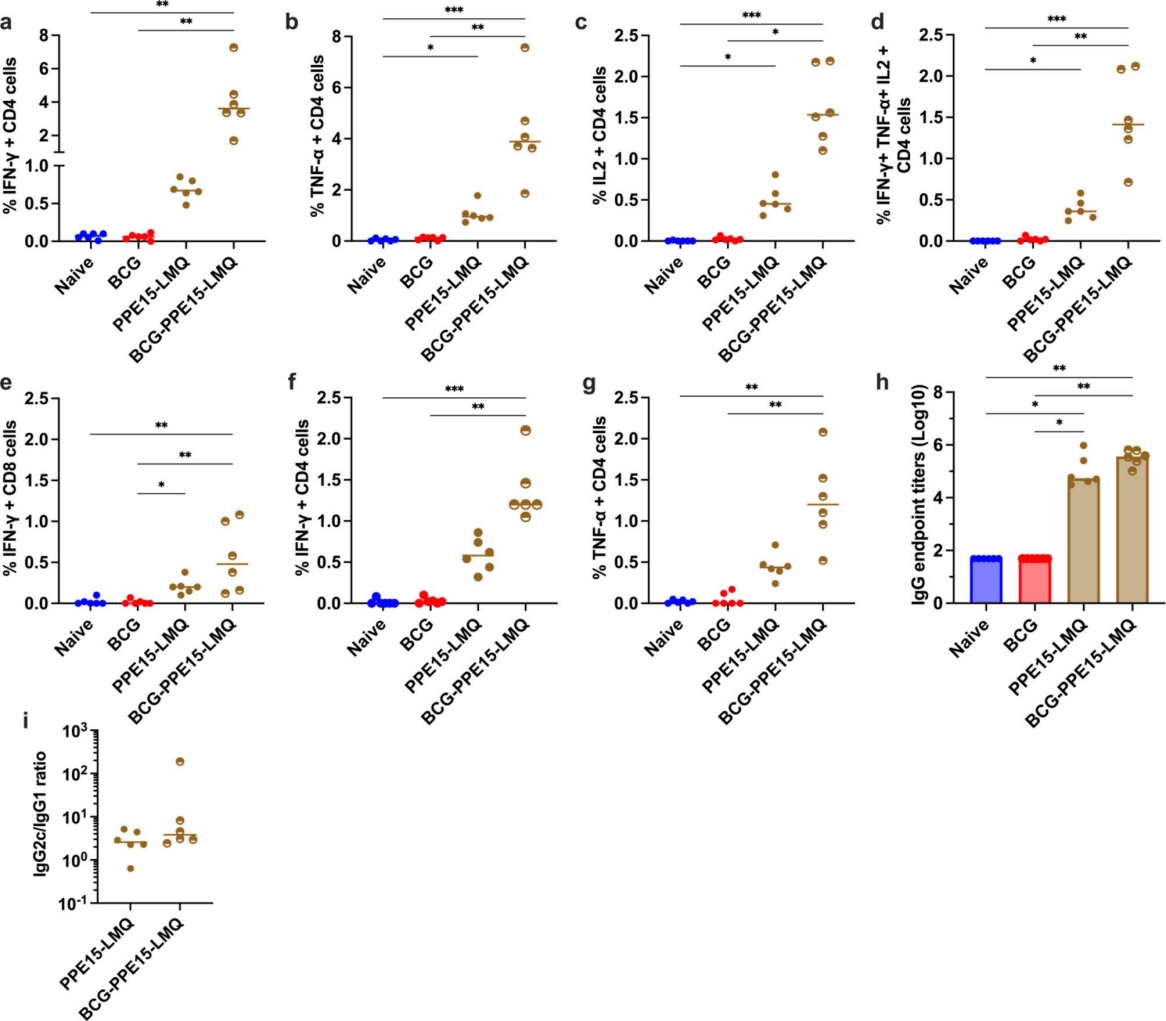
Cellular and humoral responses following vaccination with PPE15-LMQ in BCG-primed mice. Animals were primed with BCG or left unvaccinated and boosted with two doses of PPE15-LMQ. Immune responses were quantified four weeks after the last vaccination. The graph illustrates the proportion of CD4 + T cells expressing (**a**) IFN-γ, (**b**) TNF-α, (**c**) IL-2, or (**d**) triple positive cells. Proportion of CD8 + T cells that are (**e**) IFN-γ + in the spleen. Proportion of CD4 + cells expressing (**f**) IFN-γ, (**g**) TNF-α in the lung. (**h**) Serum samples were collected to assess PPE15 IgG responses (starting dilution 1:100) (**i**) and the ratio of IgG2c to IgG1. Each symbol in the graphs represents an individual animal, *n* = 6 per group. The lines denote the median value for each group. Statistical analyses were performed using the Kruskal-Wallis test, followed by Dunn’s multi-comparison test to evaluate differences between groups. Significance is denoted as follows: *p < 0.05, **p < 0.01, ***p < 0.001. Data are representative of two independent experiments.

To identify immune responses associated with the protection observed in the BCG-PPE15-LMQ group, cellular and antibody responses were assessed four weeks after the last vaccination. Enhanced CD4 + T-cell responses, including significantly higher levels of IFN-γ-, TNF-α−, IL-2-, and triple-positive cells were observed in the BCG-PPE15-LMQ group compared to both BCG-vaccinated and naïve mice (Fig. 4a–d). Although there was a trend towards higher responses in the BCG-PPE15-LMQ group compared to PPE15-LMQ alone, this did not reach significance. IFN-γ-secreting CD8 + T-cells were observed in both the PPE15-LMQ and BCG-PPE15-LMQ groups, with a trend toward stronger responses in the latter (Fig. 4e).

We next investigated the immune responses induced in the lung. Despite the systemic administration of all vaccines, there were detectable CD4 + T cell IFN-γ and TNF-α responses in both PPE15-LMQ and BCG-PPE15-LMQ groups, indicating an effective induction of immune responses in the lung (Fig. 4f, g). Responses in the BCG-PPE15-LMQ group were significantly better compared to naïve and BCG control groups (Fig. 4f, g). Strong PPE15-specific antibodies and a similar IgG2c/IgG1 ratio were detectable in the serum of PPE15-LMQ vaccinated groups (Fig. 4h, i).

### Induction and characterisation of localised immune responses by PPE15-LMQ vaccination

**Fig. 5:**
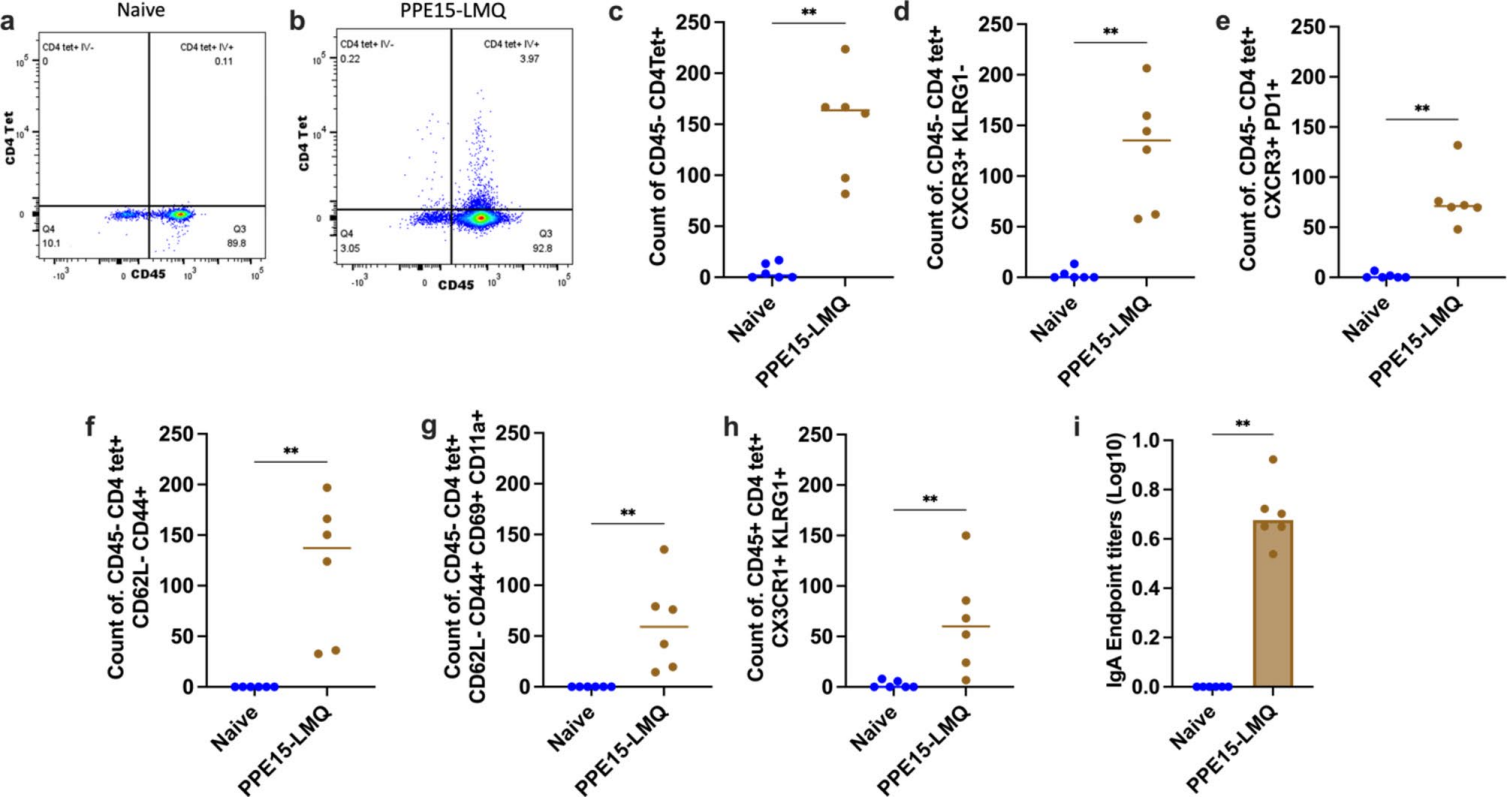
Characterisation of lung immune responses in PPE15-LMQ vaccinated animals. Four-weeks following vaccination with two doses of i.m. PPE15-LMQ, in vivo intravascular staining with αCD45 was conducted followed by in vitro tetramer and surface straining, to identify PPE15-specific cells. (**a**,** b**) Representative plots of I-A(b) PPE15 1–15 tetramer and intravascular staining of CD4 + T cells. (**c**) Number of tetramer-positive and intravascular staining-negative CD4 + T cells that are (**d**) CXCR3 + and KLRG1−, (**e**) CXCR3 + and PD1+, (**f**) effector memory (CD62L− and CD44+), (**g**) resident memory (CD62L−, CD44+, CD69 + and CD11a+). (**h**) Number of tetramer-positive and intravascular staining-positive CD4 + T cells that are CX3CR1 + KLRG1+. (**i**) BAL fluid was used to measure PPE15-specific IgA using ELISA. Each symbol in the graphs represents an individual animal, *n* = 6 per group. The lines represent median value for each group. Statistical analyses were performed using Mann–Whitney test. Significance is denoted as follows: **p < 0.01. Data are representative of two independent experiments.

Resident memory T cells (TRM) residing in the lung have been associated with protection in several TB studies^13,28,29^. Following the observation that PPE15-LMQ can induce lung responses, we wanted to determine whether PPE15-LMQ is able to induce antigen-specific TRM. We therefore utilised intravascular staining, to discriminate cells located in the lung parenchyma from cells in the vasculature^13^. To identify antigen specific cells, we used a I-A(b) PPE-15 tetramer (tet). CD45− CD4tet+ (intravascular staining protected) are PPE15-specific cells in the lung parenchyma vs. CD45 + CD4tet + that are in the vasculature. CD4tet + cells were absent in the naïve group (Fig. 5a) but were present in PPE15-LMQ vaccinated mice (Fig. 5b). CD4tet + T cells protected from intravascular staining were predominantly characterised by expression of the adhesion molecule CXCR3 + and low expression of KLRG1- (CXCR3^hi^KLRG1^low^) (Fig. 5c, d). KLRG1, a marker associated with terminal differentiation, was previously shown to be absent in cells in the parenchyma but present in cells in the lung vasculature^28^.

Cells in the parenchyma also expressed programmed cell death protein 1 (PD-1), a marker commonly co-expressed by CXCR3 + lung parenchymal cells and previously associated with protection against *M. tb* infection^28^ (Fig. 5e). Further characterisation of lung parenchymal PPE15-specific cells revealed a predominance of T-effector memory phenotype (CD44 + CD62L−), with a majority expressing T-cell resident memory marker CD69, which limits tissue egress, and the adhesion molecule CD11a^30^ (CD69 + CD11a+) (Fig. 5f, g). A significant number of PPE15-specific CD4 + T cells were also identified in the lung vasculature and were CX3CR1 + KLRG1+ (Fig. 5h). Cells with CX3CR1 + KLRG1 + phenotype were not found in CD45− cells (Supplementary Fig. 6). Additionally, PPE15-specific IgA was also detected in the BAL fluid of vaccinated animals (Fig. 5i).

### Improved immune responses and protection following heterologous vaccination with ChAdOx1.PPE15 and PPE15-LMQ

**Fig. 6:**
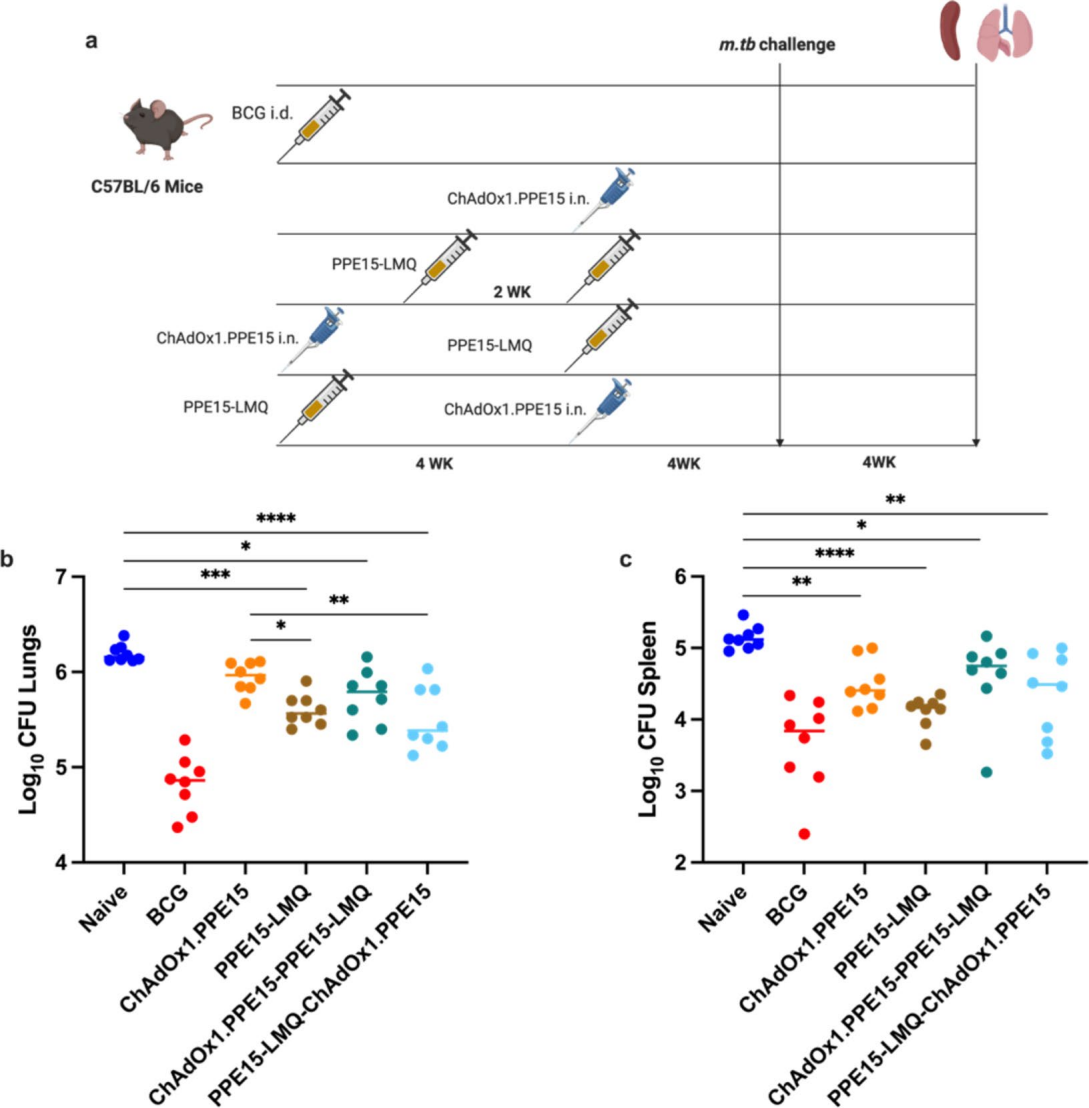
Comparison of the protective efficacy of heterologous vaccination regimens compared to homologous or single vaccinations. (**a**) Experimental schema (created with BioRender.com). (**b**) Lungs and (**c**) spleens were harvested for colony-forming unit (CFU) enumeration four weeks post-challenge. Each dot represents an individual animal, *n* = 8 per group. Experiment completed once. The lines indicate the median CFU count for each group. Statistical analyses were performed using the Kruskal-Wallis test, followed by Dunn’s multi-comparison test to evaluate differences between groups. *p < 0.05, **p < 0.01,****p < 0.0001. *CFU* colony forming units, *i.d.* intradermal, *i.m.* intramuscular.

Previous experiments demonstrated the protective potential of ChAdOx1.PPE15, when administered intranasally (i.n.)^13^. We next tested whether a heterologous vaccination with i.n. ChAdOx1.PPE15 and i.m. PPE15-LMQ could provide comparable or improved protection relative to homologous prime-boost vaccination with PPE15-LMQ. Since there is evidence to suggest that systemic priming, followed by intranasal boosting, can increase the number of antigen-specific cells in the lung, an effect known as prime-pull^31,32^, we also tested whether the order of delivery affected the outcome. In this instance, i.n. ChAdOx1.PPE15 was administered either before or after i.m. PPE15-LMQ (Fig. 6a) and groups of mice receiving either a single vaccination of ChAdOx1.PPE15 or homologous PPE15-LMQ were included. Four weeks after the last vaccination, all animals were infected with *M. tb* to assess regimen efficacy.

Following challenge, BCG-vaccinated animals had a reduced lung and spleen bacterial load compared to the unvaccinated control group (Fig. 6b, c). There was a trend for a reduced CFU in animals vaccinated with ChAdOx1.PPE15 compared to unvaccinated control (*p* = 0.0516). Homologous vaccination with PPE15-LMQ significantly reduced the lung bacterial load compared to naïve and ChAdOx1.PPE15 vaccinated mice. Both heterologous prime-boost combinations reduced lung CFU compared to the unvaccinated control group, with a trend for lower bacterial load in the PPE15-LMQ—ChAdOx1.PPE15 compared to ChAdOx1.PPE15—PPE15-LMQ (Fig. 6b). In the spleen, significant reduction in bacterial load was observed in all vaccinated groups (Fig. 6c).

Previous data suggested that ChAdOx1.PPE15 induced strong CD4+ and higher CD8+ T cell responses^13^. In contrast, responses induced by PPE15-LMQ were more skewed toward CD4+ T cell induction. To test whether combining ChAdOx1.PPE15 with PPE15-LMQ would result in a balanced CD4+ and CD8+ T cell response, we compared the immune responses of the homologous and heterologous regimens, and with ChAdOx1.PPE15 alone.

**Fig. 7:**
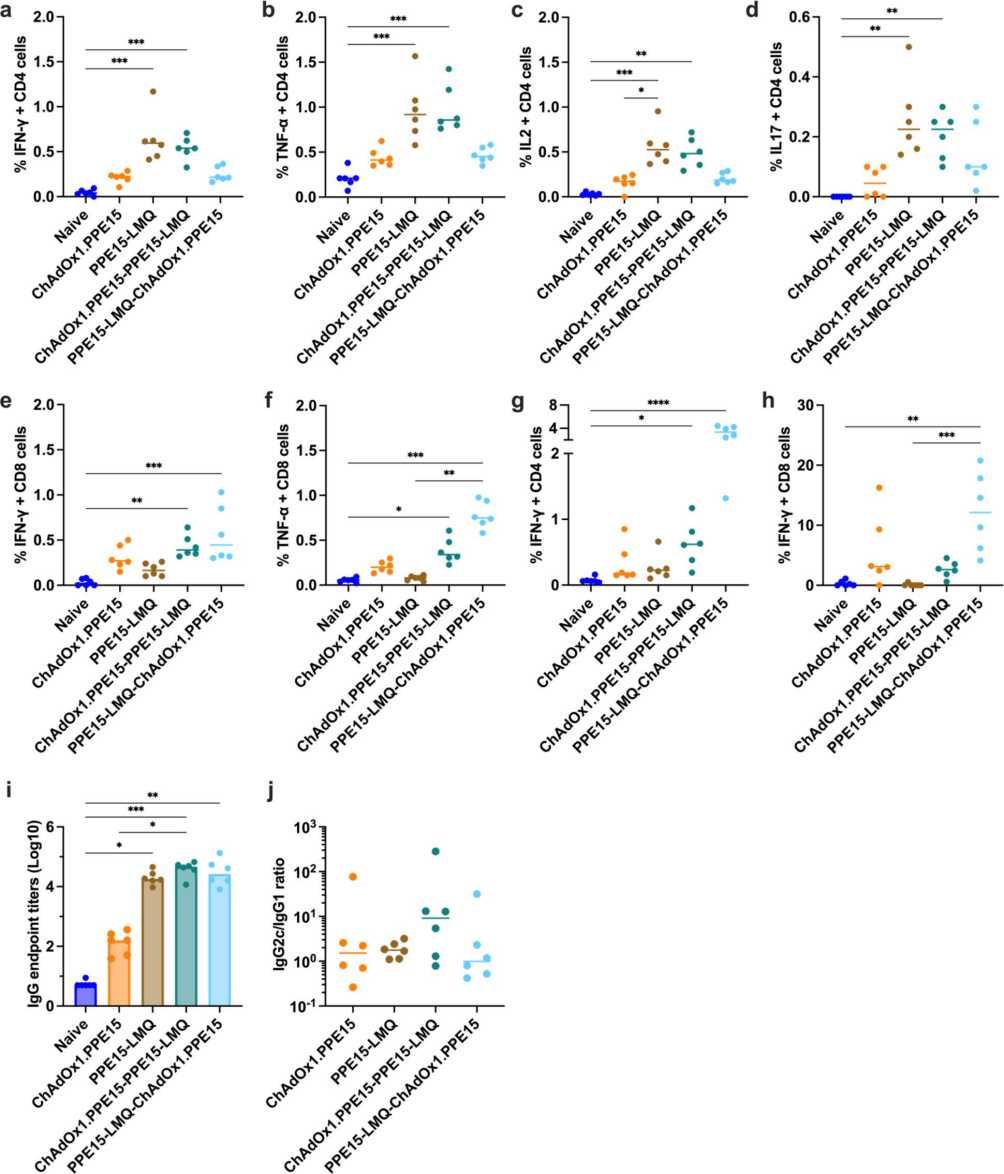
Cellular and humoral responses following heterologous vaccination regimes. Four weeks after the last immunisation, splenocytes and lung cells were harvested and stimulated with PPE15 peptide pool. Proportion of CD4 + T cells expressing (**a**) IFN-γ, (**b**) TNF-α, (**c**) IL-2, (**d**) IL-17 and proportion of CD8 + T cells (**e**) IFN-γ and (**f**) TNF-α positive in the spleen. Proportion of (**g**) CD4 + and (**h**) CD8 T cells expressing IFN-γ in the lung. (**i**) Serum PPE15-specific IgG endpoint titres (starting dilution 1:10) (**j**) and the ratio of IgG2c to IgG1. Each symbol in the graphs represents an individual animal, *n* = 6 per group. The lines represent median value for each group. Statistical analyses were performed using the Kruskal-Wallis test, followed by Dunn’s multi-comparison test *p < 0.05, **p < 0.01, ***p < 0.001, ****p < 0.0001. Data are representative of two independent experiments.

In the spleen, ChAdOx1.PPE15—PPE15-LMQ induced comparable CD4 + T cell responses to the PPE15-LMQ group, despite a single i.m. vaccination (Fig. 7a–d). CD8 + T cell responses (IFN-γ and TNF-α) were detectable across groups but were higher in ChAdOx1.PPE15—PPE15-LMQ and PPE15-LMQ—ChAdOx1.PPE15 compared to naïve mice (Fig. 7e, f).

In the lungs, all vaccinated groups had a CD4 + T cell IFN-γ response following PPE15-stimulation. Both heterologous groups had significantly higher response compared to naïve mice (Fig. 7g). CD8 + T cell IFN-γ responses were observed only in groups that received ChAdOx1.PPE15 vaccination. Samples from the PPE15-LMQ—ChAdOx1.PPE15 group had significantly higher responses compared to both the naïve and PPE15-LMQ groups (Fig. 7h). Antigen-specific CD4+ and CD8+ T cells IFNγ responses were predominantly localised in the lung parenchyma (Supplementary Fig. 7).

Antigen-specific IgG responses were detectable in all groups. Combining ChAdOx1.PPE15 with PPE15-LMQ improved ChAdOx1.PPE15 antibody responses. Similar IgG2c/IgG1 ratio were detectable in the serum (Fig. 7i, j).

**Fig. 8:**
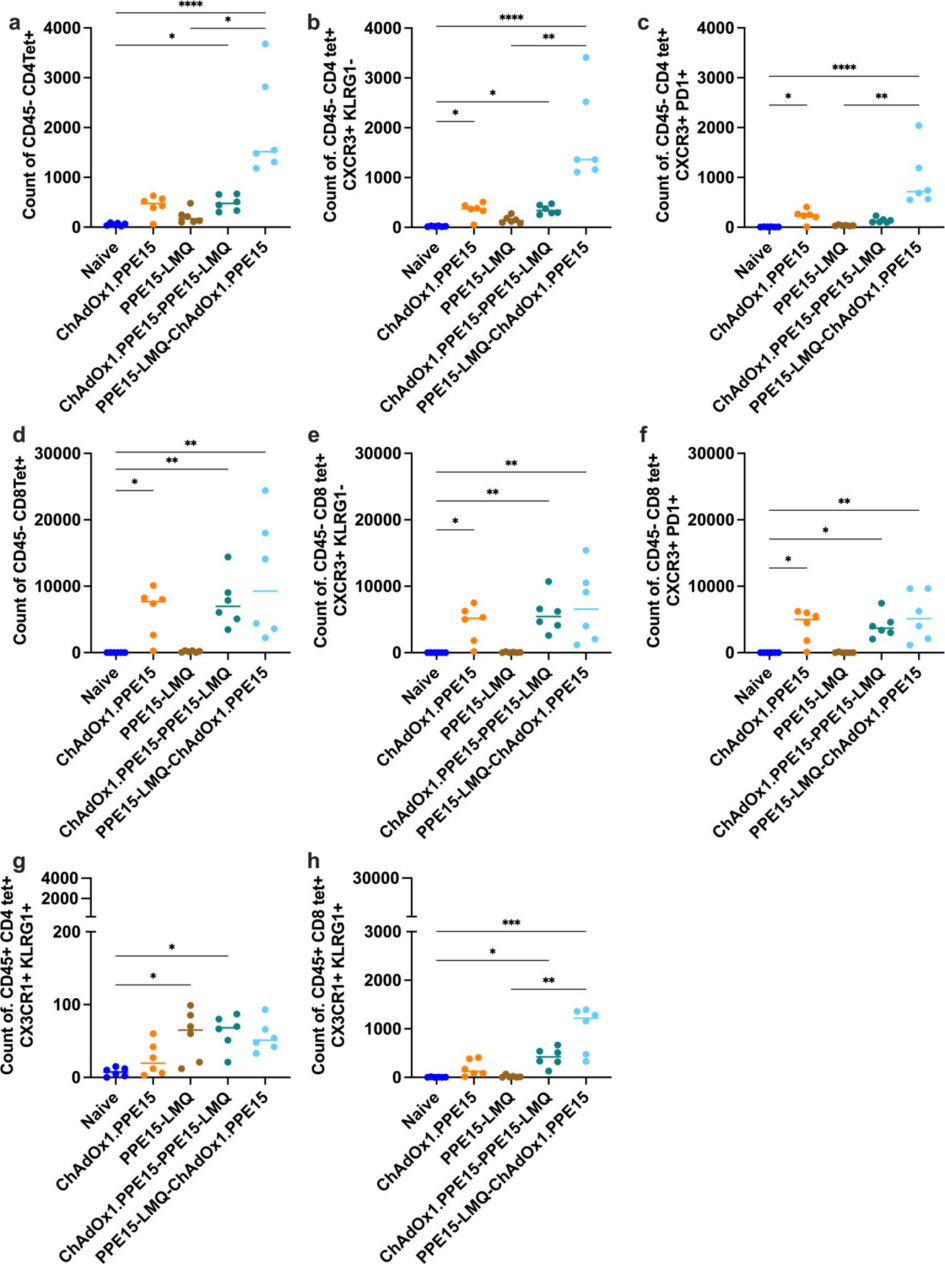
Characterisation of the phenotype of antigen-specific T cells in the lung parenchyma and vasculature of heterologous vaccination regimes. Four weeks after the last vaccination, PPE15 I-A(b) and H-2D(b) tetramers were combined with intravascular staining with αCD45. Number of tetramer-positive and intravascular staining-negative CD4 + T cells (**a**) that are (**b**) CXCR3 + and KLRG1− (**c**) CXCR3 + and PD1+. Number of tetramer-positive and intravascular staining-negative CD8 + T cells (**d**) that are (**e**) CXCR3 + and KLRG1− (**f**) CXCR3 + and PD1+. Number of tetramer-positive and intravascular staining-positive (**g**) CD4 + and (**h**) CD8 + T cells that are CX3CR1 + KLRG1+. Each dot represents one animal, *n* = 6 per group. Experiment completed once. The lines represent median value for each group. Statistical analyses were performed using the Kruskal-Wallis test, followed by Dunn’s multi-comparison test to evaluate differences between groups. Significance is denoted as follows: *p < 0.05, **p < 0.01, ***p < 0.001, ****p < 0.0001.

To further characterise lung responses following vaccination with the heterologous regimens, PPE15 I-A(b) and H-2D(b) tetramers were combined with intravascular staining with an αCD45 antibody. The PPE15-LMQ—ChAdOx1.PPE15 regimen induced a significant population of intravascularly protected CD4tet + T cells, which predominantly exhibited a resident memory phenotype (tet + CXCR3 + KLRG1− and tet + CXCR3 + PD1+) compared to naïve and PPE15-LMQ groups (Fig. 8a–c).

ChAdOx1.PPE15 and heterologous prime-boost groups induced PPE15-specific CD8 + T cells in the lung parenchyma with resident memory phenotype (CXCR3 + KLRG1− and CXCR3 + PD1+) (Fig. 8d–f).

The heterologous groups had comparable antigen-specific CD4+ (Fig. 8g) and CD8 + T cells in the lung vasculature (CD45+), with a trend toward higher CD8 + T cell responses in the PPE15-LMQ—ChAdOx1.PPE15 group (Fig. 8h).

### BCG boosting effect of ChAdOx1.PPE15 and PPE15-LMQ regimes

**Fig. 9:**
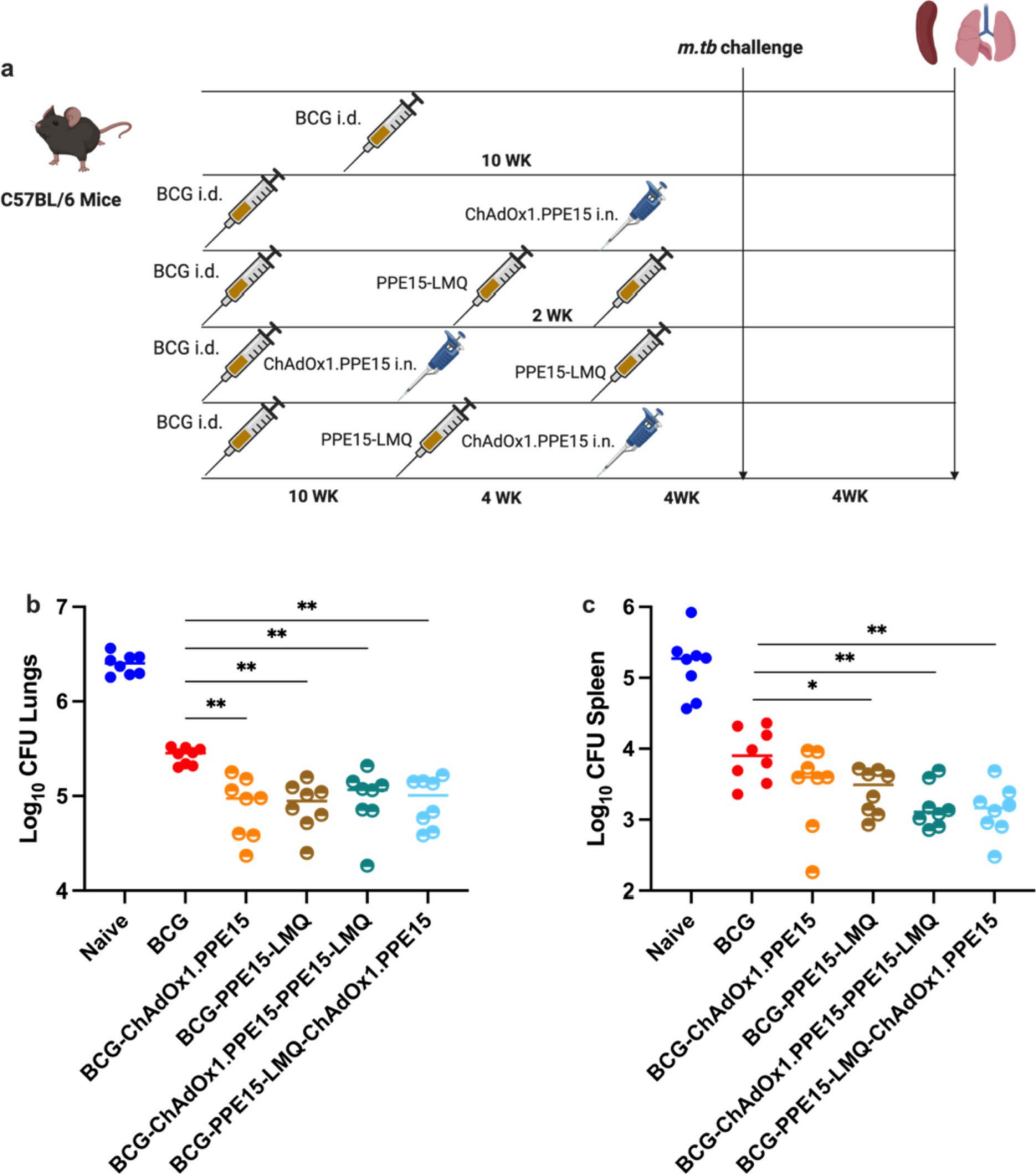
Comparison of protective efficacy of heterologous vaccination regimens compared to homologous or single vaccination regimes on BCG-Primed mice. (**a**) Experimental schema (created with BioRender.com). (**b**) Lungs and (**c**) spleens were harvested for colony-forming unit (CFU) enumeration four weeks post-challenge. Each dot in the figures represents an individual animal, and the lines indicate the median CFU count for each group, *n* = 8 per group. Experiment completed once. Statistical analyses were performed using the Kruskal-Wallis test, followed by Dunn’s multi-comparison test to evaluate differences between groups. *p < 0.05, **p < 0.01. *CFU* colony forming units, *i.d.* intradermal, *i.m.* intramuscular.

As these vaccine candidates are all designed as subunit BCG booster vaccines for clinical translation, it was important to determine the ability of the different vaccine candidates to improve the efficacy of BCG vaccination. To test this, mice were primed with BCG and boosted with either i.n. ChAdOx1.PPE15 or PPE15-LMQ. Heterologous regimens combining the two platforms were also tested, as above (Fig. 9a).

All vaccine regimens provided protection in the lungs compared to naïve, and all improved the efficacy of BCG, with each group demonstrating similar efficacy (Fig. 9b). In the spleen, all vaccine regimens resulted in a significant CFU reduction compared to the naïve group. All vaccination regimens except ChAdOx1.PPE15 also improved the efficacy of BCG (Fig. 9c).

**Fig. 10:**
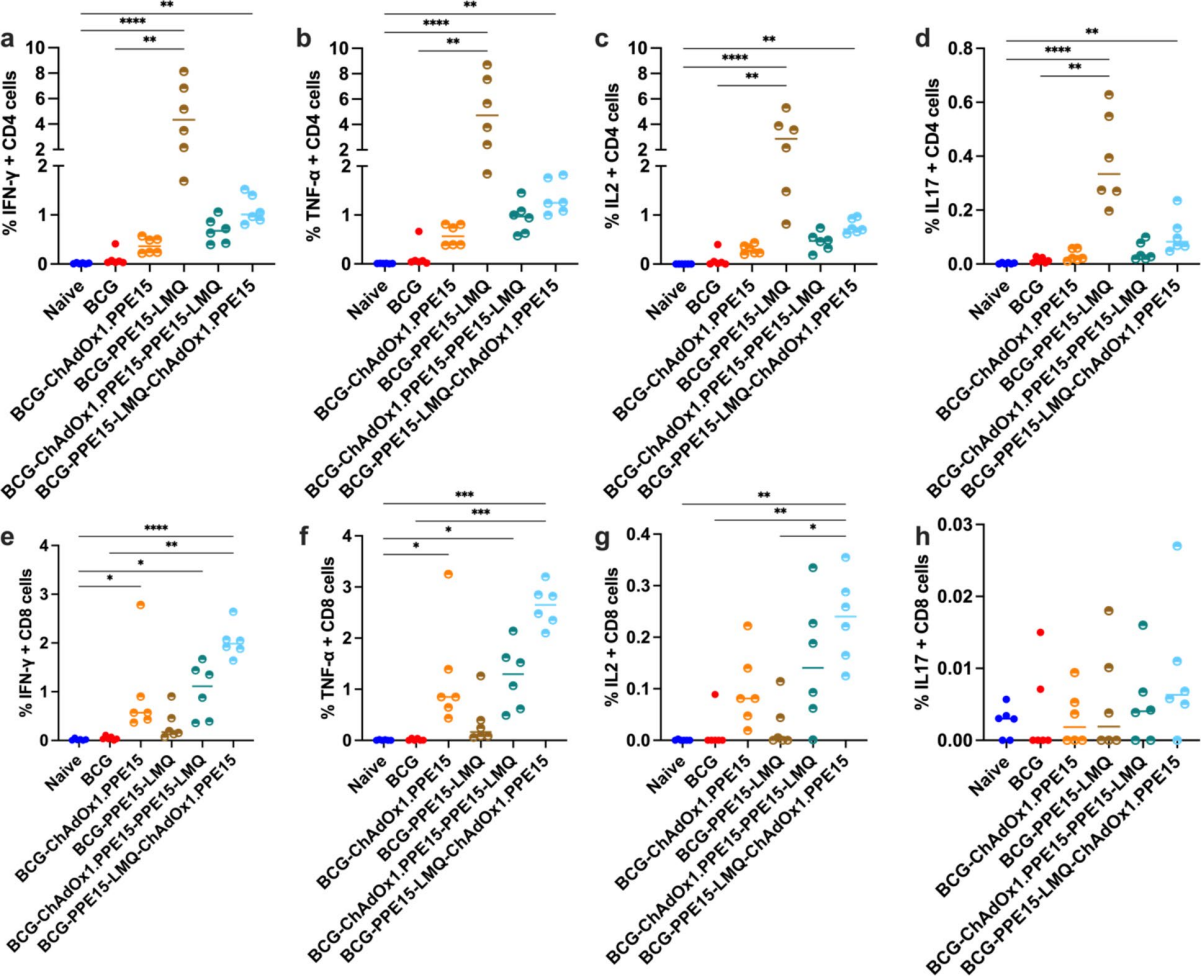
Cellular responses induced following vaccination by heterologous vaccination approach of intranasal ChAdOx1.PPE15 and PPE15-LMQ on BCG-primed mice. Mice were vaccinated as in Fig. 9a. Four weeks after the last vaccination, splenocytes were harvested and vaccine immunogenicity was determined. Proportion of CD4 + T cells (**a–d**) and CD8 + T cells (**e–h**) that are positive for (**a**,** e**) IFN-γ, (**b**,** f**) TNF-α, (**c**,** g**) IL-2, and (**d**,** h**) IL-17. Each symbol in the graphs represents an individual animal, and the lines denote the median response for each group, *n* = 6 per group. Experiment completed once. Statistical analyses were performed using the Kruskal-Wallis test, followed by Dunn’s multi-comparison test to evaluate differences between groups. Significance is denoted as follows: *p < 0.05, **p < 0.01, ***p < 0.001, ****p < 0.0001.

Comparison of systemic immune responses indicated that both BCG-PPE15-LMQ and BCG-PPE15-LMQ—ChAdOx1.PPE15 induced higher CD4 + IFN-γ, TNF-α, IL-2, and IL-17 responses compared to the naïve and BCG groups, with a trend toward the strongest responses in the BCG-PPE15-LMQ group (Fig. 10a–d). In contrast to CD4 + responses, BCG-PPE15-LMQ did not induce a strong CD8 + T cells response. However, combination with ChAdOx1.PPE15 resulted in robust CD8 + IFN-γ and TNF-α responses in BCG-PPE15-LMQ and BCG-primed heterologous regimens (Fig. 10e, f). CD8 + IL-2 + cells were also higher in the BCG-PPE15-LMQ - ChAdOx1.PPE15 group compared to the naïve, BCG, and BCG-PPE15-LMQ groups (Fig. 10g). Low CD8 + IL-17 + cells on all groups (Fig. 10h).

**Fig. 11:**
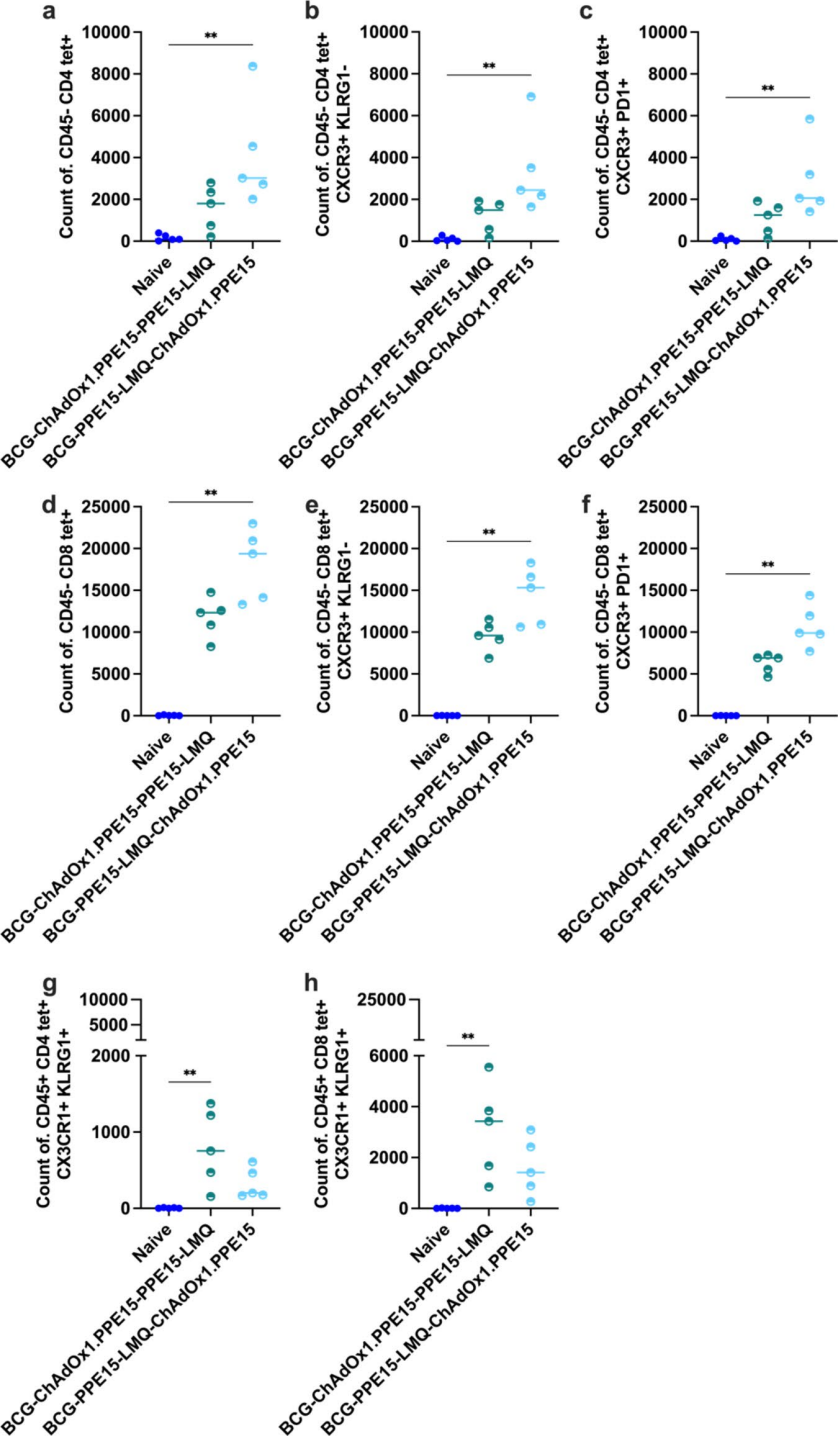
Characterisation of the phenotype of antigen-specific T cells in the lung parenchyma and vasculature of heterologous vaccination regimes. Four weeks after the last vaccination, PPE15 I-A(b) and H-2D(b) tetramers were combined with intravascular staining with αCD45. Number of tetramer-positive and intravascular staining-negative CD4 + T cells (**a**) that are (**b**) CXCR3 + and KLRG1− (**c**) CXCR3 + and PD1+. Number of tetramer-positive and intravascular staining-negative CD8 + T cells (**d**) that are (**e**) CXCR3 + and KLRG1− (**f**) CXCR3 + and PD1+. Number of tetramer-positive and intravascular staining-positive (**g**) CD4 + and (**h**) CD8 + T cells that are CX3CR1 + KLRG1+. Each dot represents one animal, and the lines represent the median value for each group, *n* = 5 per group. Experiment completed once. Statistical analyses were performed using the Kruskal-Wallis test, followed by Dunn’s multi-comparison test to evaluate differences between groups. Significance is denoted as follows: **p < 0.01.

To determine the capacity of the heterologous prime-boost regimens to induce TRM in BCG-primed mice, we primed mice with BCG, which we then boosted with either ChAdOx1.PPE15 - PPE15-LMQ or PPE15-LMQ - ChAdOx1.PPE15. Both regimens induced CD4 + T cells with resident memory phenotype (tet + CXCR3 + KLRG1− and tet + CXCR3 + PD1+), with PPE15-LMQ - ChAdOx1.PPE15 significantly higher compared to naïve group (Fig. 11a–c). Heterologous prime-boost groups also induced CD8 + T cells in the lung parenchyma with resident memory phenotype (CXCR3 + KLRG1− and CXCR3 + PD1+) (Fig. 11d–f). Both groups had antigen-specific CD4 + and CD8 + T cells in the lung vasculature (CD45+) with a trend for higher responses in the ChAdOx1.PPE15-PPE15-LMQ group (Fig. 11g, h).

## Discussion

TB vaccine development is challenging due to the complexity of *M. tb* pathogenesis and the lack of well-defined correlates of protection^33,34^. Unlike other diseases, TB vaccine efficacy trials are long, costly, and require large-scale population studies with extended follow-up^35^. Additionally, limited funding for TB research further hinders progress^36,37^.

Despite these challenges, recent clinical trials, M72/AS01E, indicate that inducing protective immunity against TB is feasible^7^. Furthermore, these findings highlight the potential of protein-adjuvanted subunit vaccines, particularly if paired with scalable, cost-effective solutions, essential for low- and middle-income countries (LMICs), where TB burden is highest^38,39^. To address this, we partnered with VFI to access adjuvants, designed for open-access technology transfer and local manufacturing^15^. One of these, LMQ, has similar immunomodulatory components to AS01^40^. Our selection of PPE15 as a model antigen was based on its broad and common recognition in active and latently infected individuals^11,12^, as well as its protective efficacy in mice^13,14^. This work highlights the potential of PPE15-LMQ for use as a Prevention of Infection (POI) vaccine.

We demonstrated that whilst saponin-containing adjuvants were immunoenhancing, LMQ was the most promising as it induced a robust CD4 + T cell response both in the lung and spleen and provided protection against *M. tb* infection, both as a standalone vaccine and as a booster to BCG. In addition, we demonstrated that combination of PPE15-LMQ with ChAdOx1.PPE15 was also protective.

The pronounced CD4 + IFN-γ, TNF-α, IL-2, responses elicited by PPE15-SQ, -SMQ, -LQ, and -LMQ formulations are a critical part of an effective Th1 response and are essential for protection against *M. tb*^41–45^. Notably, in the absence of the CD4 + subset or Th1-type cytokines, mice succumb to *M. tb* infection early, with markedly high bacterial loads^46–48^. Similarly, HIV-infected patients with CD4 + cell deficiency show increased reactivation of latent *M. tb* infections and altered TB disease pathology, marked by diffuse necrotic lesions rather than the structured granulomas typically seen in immune-competent individuals^49^. In addition to promoting Th1 responses, SQ, SMQ, LQ and LMQ adjuvants induced IL-17, a cytokine linked to protective immunity against *M. tb*^50,51^. Studies indicate that IL-17 knockout (KO) mice exhibit higher bacterial loads and diminished granuloma formation, both of which are crucial for effective immunity^52,53^. Furthermore, the neutralisation of IL-17 with antibodies partially reduced the protective effect provided by vaccination, suggesting IL-17’s essential role^51^. A potential mechanism behind this protection is the ability of IL-17 to recruit neutrophils to the sites of infection during the acute phase, enhancing immune defence^54^. Moreover, the induction of polyfunctional CD4 + T cells, characterised by their simultaneous production of IFN-γ, TNF-α, and IL-2, suggests a robust and multifaceted adaptive immune response. These cells are known for their superior effector functions and have been associated with protection from *M. tb* infection in animal and human models^22,55^. These polyfunctional CD4 + T cells were significantly more frequent in mice vaccinated with PPE15-LMQ and PPE15-SMQ, highlighting the robust immune activation achieved by combining QS21 and TLR4 agonists. These findings align with the latest clinical trial on M72/AS01_E_^7^, in which the M72 fusion protein is formulated with AS01_E_, composed of liposomes containing the same class of immunomodulators as LMQ^40,56,57^. In contrast, the low Th1 responses observed with PPE15-SWE serve as proof-of-concept that the choice of adjuvant is as crucial as the antigen itself in inducing a robust immune response. Squalene exerts immunostimulatory effects by recruiting macrophages and dendritic cells (DCs) to the injection site through chemokine secretion via an NLRP3-independent apoptosis-associated speck-like protein (ASC) activation pathway and a TLR-independent MyD88 activation pathway. In the absence of QS21 and TLR4 ligand, this leads to a Th2-biased immune response^58^, as demonstrated by the ratio of IgG2c/IgG1 calculated as lower than 1 (Fig. 2h).

Adding QS21 to squalene in the SQ formulation resulted in a bias toward Th1 response (Fig. 2h). QS21 (Q) is a triterpene glycoside purified from the bark extracts of the *Quillaja saponaria* Molina tree. It triggers the NLRP3 inflammasome and the subsequent release of caspase-1-dependent cytokines, IL-1β and IL-18, which are important for Th1 responses^59^. The further addition of 3D6AP (M) in the formulation further improved Th1 responses. 3D6AP is a synthetic TLR4 agonist that resembles bacterial monophosphoryl lipid A, and can induce Th1-biased immune responses^60^.

Liposomes made of DOPC have also demonstrated efficacy as adjuvant components by serving as a delivery system to antigen-presenting cells (APCs). They protect antigens from premature degradation and maintain ingredients with prolonged release, thereby enhancing exposure to the antigen and immunostimulators^61,62^. In our immunogenicity study, squalene and liposome formulations induced Th-1 responses that were comparable when QS21 was present, but there was a trend for liposomes to be superior when both QS21 and 3D6AP were present. All adjuvants induced a robust antibody response, but there was a noticeable increase in the ratio of IgG2c/IgG1 when 3D6AP was included. The role of antibodies in TB is more complex than initially appreciated, and studies support a protective role. Studies in non-human primates using mucosal or intravenous BCG have shown near-sterilising immunity to *M. tb* infection challenge, which correlated with increased *M. tb*-specific IgG, IgA, and IgM antibody levels in the plasma and BAL fluid^26,63^. Notably, the IgG2c/IgG1 ratio suggested a skew towards a Th1-type immune response^64,65^. These protein-adjuvanted vaccines have a weak ability to induce CD8 + T cell-mediated cellular immunity, which aligns with current adjuvant literature^58^ and findings from the M72/AS01 trial^7^.

Using a mycobacterial growth inhibition assay (MGIA) as a screening tool, we identified PPE15-LMQ and PPE15-SQ as promising candidates. This approach allowed us to narrow down the candidates and reduce the number of animals needed for *M. tb* challenge studies^66^. However, since the MGIA does not fully replicate the complexity of in vivo immune responses, we next confirmed the efficacy of PPE15-LMQ in in vivo *M. tb* challenge studies. Our data further support the use of MGIA in the early evaluation of TB vaccine candidates^24^. Specific lung immunity at the site of infection, along with a robust systemic immune response, are important contributors in protection against pulmonary TB^67^. We showed that despite its systemic administration, PPE15-LMQ induced a robust lung CD4 + response and PPE15-specific IgA in the BAL. Lung responses had a TRM phenotype associated with protection during TB challenge^28^ and with protective TB vaccines^13,68^. These findings align with previous results showing that in BCG-primed rhesus macaques, boosted with M72/AS01_E_ increased peptide-specific CD4 + T cells in the BAL after the first (*p* < 0.03) and second (*p* < 0.05) boost, compared with pre-boost (Week 12)^69^.

Heterologous vaccination strategies, which use different vaccine platforms for the prime and boost doses, have been shown to enhance immune responses by leveraging the strengths of each platform and by inducing a broader and more robust immune response^70–72^. We aimed to further improve TRM generation by utilising a “prime-pull” strategy by priming systemically and boosting in the lung^31,32^. Mucosal delivery of vaccines might be a more successful vaccination strategy as it targets the induction of immune responses at the point of entry of *M. tb*^73^. ChAdOx1, a viral vector with natural tropism for the lung, has been shown to confer significant protection in mice^13,14,70^ and has been assessed for safety and immunogenicity in a dose-escalation phase-I clinical trial^74^. In our study, the combination of ChAdOx1.PPE15 with PPE15-LMQ showed promising results. This approach resulted in improved CD4 + T cell and antibody responses compared to ChAdOx1.PPE15 alone, and higher CD8 + responses compared to homologous prime-boost with PPE15-LMQ. These data suggest that combining protein-adjuvant with viral vector, resulted in a more balanced CD4 + and CD8 + T cell response, compared to each platform alone, and could be an alternative effective vaccination approach. Interestingly, although both heterologous combinations (PPE15-LMQ - ChAdOx1.PPE15 and ChAdOx1.PPE15 - PPE15-LMQ) improved protection in the lung and spleen, there was a trend toward lower bacterial load in the PPE15-LMQ - ChAdOx1.PPE15 group. This order of heterologous vaccination was also observed by Baldwin et al.^71^ who demonstrated that long-lived immunity to ID93 vaccines can be achieved either with an ID93/GLA-SE subunit approach or by priming with the subunit vaccine and boosting with an adenovirus Ad5-ID93 vaccine, but not the other way around. Both methods induced ID93-specific CD4 + T cell responses, but only the heterologous approach induced ID93-specific CD8 + T cell responses. Bouillet et al.^75^ demonstrated that the sequence of heterologous immunisations can significantly enhance immunity using a malaria vaccine. In their study, memory T cells and antibody responses were increased when a recombinant malaria protein, administered with Montanide ISA720 adjuvant, was used as a prime, followed by an adenoviral vector vaccine as a boost. Conversely, when the adenoviral vector was used as the priming vaccine, the responses were lower. Similarly, the order of administering heterologous TB vaccines is crucial for optimal immune response. For instance, a study showed that priming with a recombinant TB protein (rMT1721) combined with GLA adjuvant, followed by a plasmid DNA vaccine expressing the same antigen, induced a more robust immune response, including both antigen-specific CD4 + and CD8 + T cells, compared to the reverse order, where CD8 + T cells were undetectable^76^. In our study, we found that heterologous prime-boost vaccinations, can induce resident memory CD4 + and CD8 + T cells and that the order of vaccination influenced the phenotype of local T cells in the lung, with PPE15-LMQ followed by ChAdOx1.PPE15 resulting in more antigen-specific resident T cells, while the reverse order induced more intravascularly localised T cells. These findings suggest that the sequence of vaccination can affect both the localisation and the phenotype of the immune response, potentially impacting the protective efficacy observed. Similar observations of this localisation impacting protective efficacy were reported with the systemic administration of AdCh68Ag85A followed by intranasal administration of Ag85 proteins. This regimen led to a significant increase in T resident memory cells and improved lung protection compared to AdCh68Ag85A alone^77^. In BCG-primed mice, although both the homologous and heterologous platforms improved BCG, the level of protection was comparable. One hypothesis is a high infection dose, or alternatively a strong BCG effect^78^. Repeating the experiment with a lower infection dose or a lower BCG dose might overcome this issue^79,80^. In addition, a longer interval between challenge and takedown might allow a better resolution between the vaccine groups and will also help to assess the long-term protective effects of our vaccines.

This study primarily focused on the induction of Th1 immunity. While we demonstrated significant enhancement of Th1 responses and lung-homing T-cells, future research should include a comprehensive analysis of innate immunity and the expansion of humoral compartments, such as lung-resident memory B cells, elicited by these vaccination regimens. These studies could help uncover the mechanisms behind the ability of LMQ-adjuvanted vaccine to induce antigen-specific lung-parenchymal immune response, despite its i.m. administration, and in the absence of a lung challenge. Furthermore, although our results suggest an indirect relationship between lung-homing T-cells and protection against *M. tb* infection, we haven’t directly demonstrated the protective role of these cells. Follow-up studies involving transfer of these cells into naïve congenic mice will help establish a direct causal link. Additionally, investigating the long-term immunity will be crucial for translating these findings into practical vaccination strategies. Our studies also demonstrated that the presence of a TLR4 agonist in the LMQ adjuvant improved Th1 responses and protection. An in-depth characterisation of the mechanisms behind the action of the individual adjuvant immunomodulators in relation to protection, would further benefit the development of suitable adjuvanted TB vaccines. Finally, while our study highlights the potential role of lung-homing T-cells in conferring protective immunity, the longevity of these cells, particularly within the context of different heterologous vaccination regimens, warrants further investigation. Understanding this and the functional stability of these cells following vaccination may be crucial for developing sustained protection against TB.

In conclusion, our study serves as an example of an in-depth evaluation to down select a suitable antigen and adjuvant combination for further development. It highlights the potential of PPE15 formulated with saponin-based adjuvants, in particular LMQ, to form an effective vaccine candidate against TB. PPE15-LMQ provided synergistic protection when used in a heterologous vaccination approach, providing a promising immunisation strategy for improving TB vaccine efficacy. In developing this vaccine candidate, we have considered the importance of accessibility, local manufacturing, technology transfer, stability, and cost-effectiveness when developing vaccines for use in low- and middle-income countries. The insights gained from this study are invaluable for the ongoing efforts to combat the global TB epidemic and could lead to a more robust and universally effective TB vaccine strategy. Notably, our data support the progression of PPE15-LMQ, alone or in combination with ChAdOx1.PPE15, to the next stage of vaccine development.

## Electronic supplementary material

Below is the link to the electronic supplementary material.

Supplementary Material 1

## Data Availability

The datasets used and/or analysed during the current study are available from the corresponding author on reasonable request.
