# Supplementary material for: Strong immune responses and robust protection following a novel protein in adjuvant tuberculosis vaccine candidate
Source: Sci Rep. 2025 Jan 13;15:1886. doi: 10.1038/s41598-024-84667-8 (PMC11729893; doi:10.1038/s41598-024-84667-8)
Supplement: Supplementary file 1 — Supplementary Material 1 [file 41598_2024_84667_MOESM1_ESM.pdf]

Supplementary Information

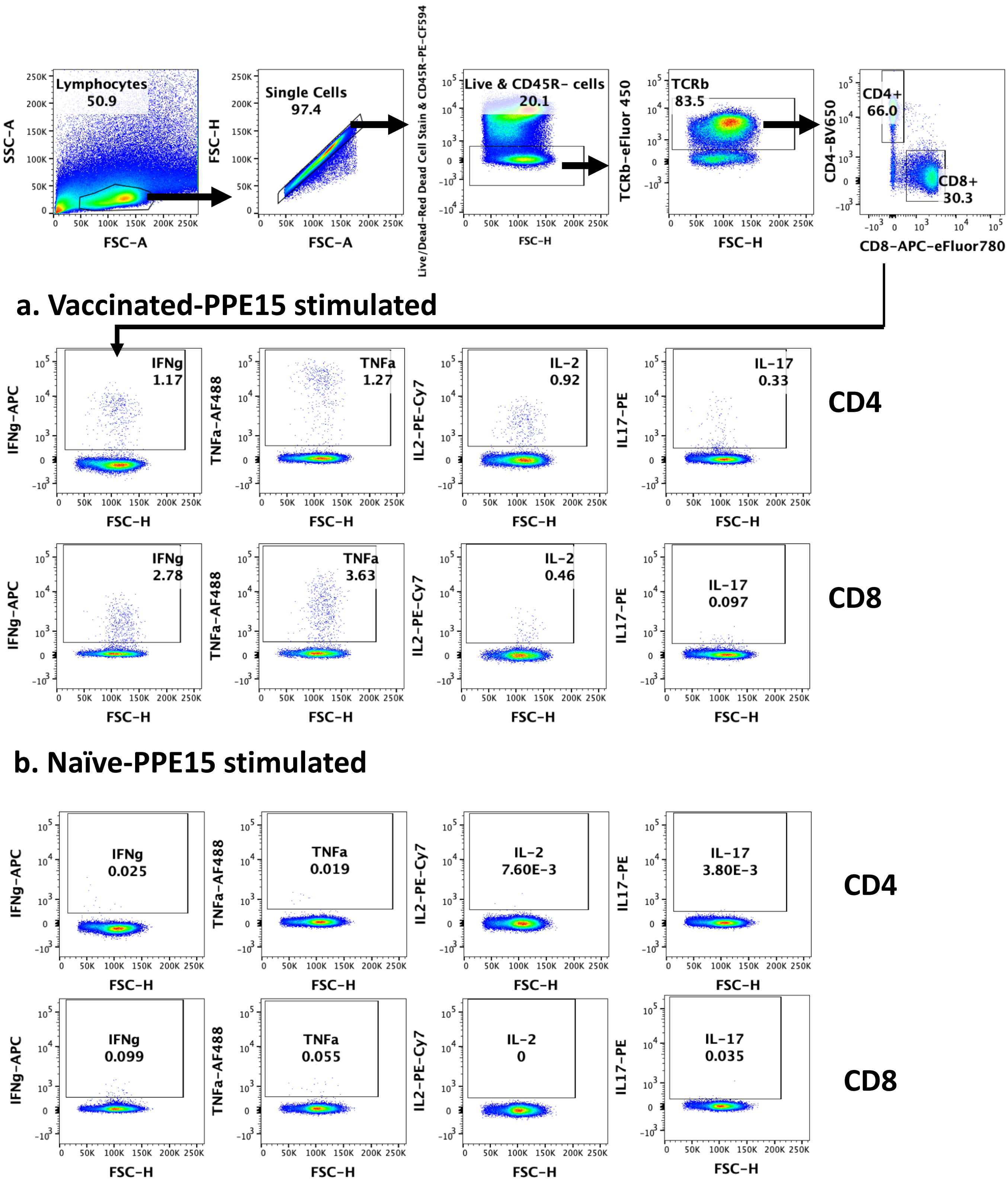

c. Vaccinated-Media

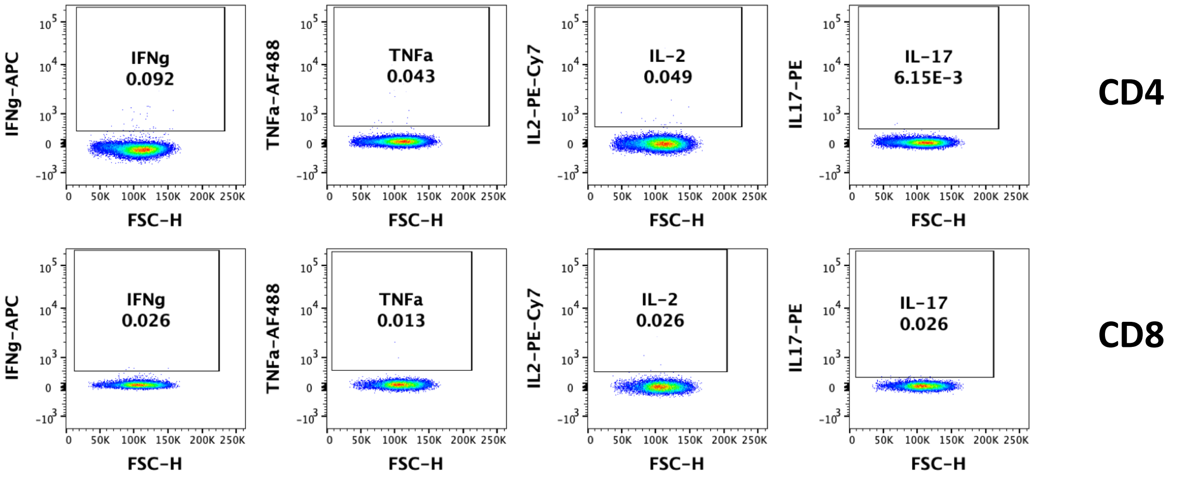

d. Naïve-Media

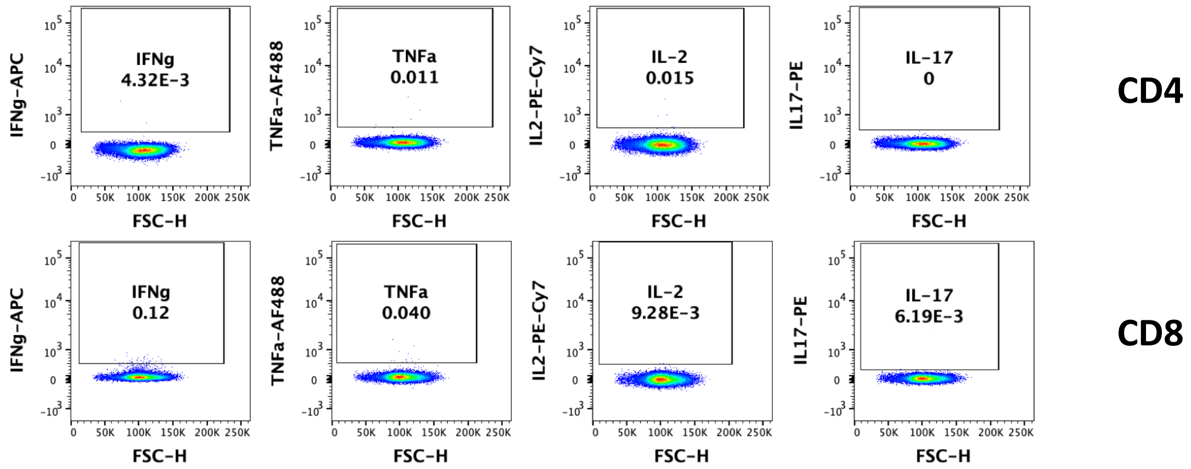

Supplementary Figure S1: Flow cytometry gating strategy for T cell surface and intracellular cytokine staining. Splenocytes from (a) vaccinated and (b) control animals were stimulated with PPE15 peptide pool to assess cytokine production. Background subtraction was performed using splenocytes from the same animals incubated with media (c, d). Lymphocytes were gated based on forward scatter (FSC) and side scatter (SSC). Following the exclusion of doublets, live CD45R- cells were selected. TCRβ+ cells were further gated into CD4+ or CD8+ subsets. Cytokine production, including IFN-γ, TNF-α, IL-2, and IL-17, was assessed for each of the CD4+ and CD8+ T cell populations.

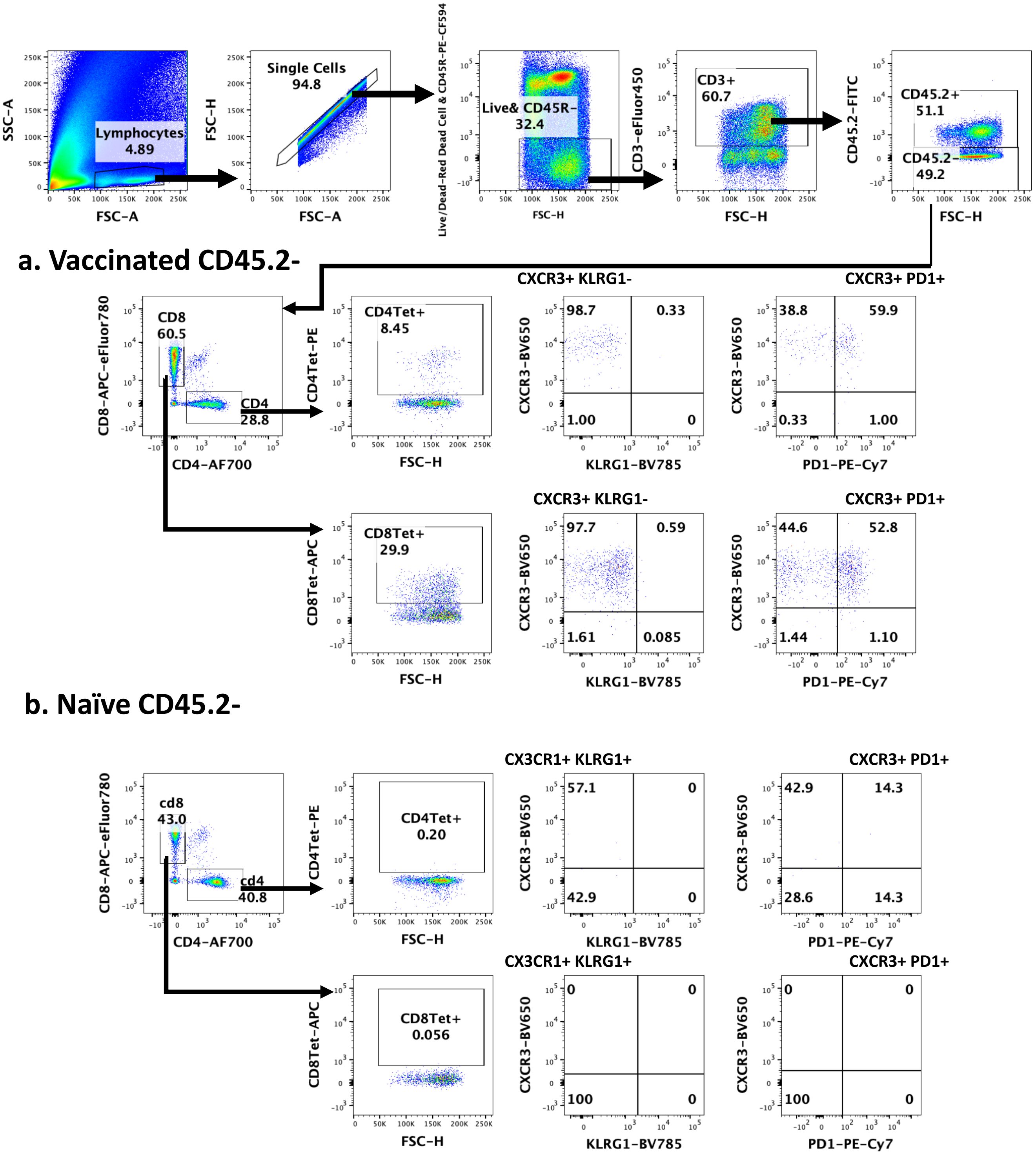

Lymphocytes
4.89
SSC-A
FSC-A
Single Cells
94.8
FSC-H
FSC-A
Live/Dead-Red Dead Cell & CD45R-PE-CF594
Live& CD45R-
32.4
FSC-H
CD3-eFluor450
CD3+
60.7
FSC-H
CD45.2-FITC
CD45.2+
51.1
CD45.2-
49.2
FSC-H
a. Vaccinated CD45.2-
CD8-APC-eFluor780
CD8
60.5
CD4
28.8
CD4-AF700
CD4Tet-PE
CD4Tet+
8.45
FSC-H
CXCR3+ KLRG1-
CXCR3-BV650
98.7
0.33
1.00
0
KLRG1-BV785
CXCR3+ PD1+
CXCR3-BV650
38.8
59.9
0.33
1.00
PD1-PE-Cy7
CD8Tet-APC
CD8Tet+
29.9
FSC-H
CXCR3+ KLRG1-
CXCR3-BV650
97.7
0.59
1.61
0.085
KLRG1-BV785
CXCR3+ PD1+
CXCR3-BV650
44.6
52.8
1.44
1.10
PD1-PE-Cy7
b. Naïve CD45.2-
CD8-APC-eFluor780
cd8
43.0
cd4
40.8
CD4-AF700
CD4Tet-PE
CD4Tet+
0.20
FSC-H
CX3CR1+ KLRG1+
CXCR3-BV650
57.1
0
42.9
0
KLRG1-BV785
CXCR3+ PD1+
CXCR3-BV650
42.9
14.3
28.6
14.3
PD1-PE-Cy7
CD8Tet-APC
CD8Tet+
0.056
FSC-H
CX3CR1+ KLRG1+
CXCR3-BV650
0
0
100
0
KLRG1-BV785
CXCR3+ PD1+
CXCR3-BV650
0
0
100
0
PD1-PE-Cy7

c. Vaccinated CD45.2+

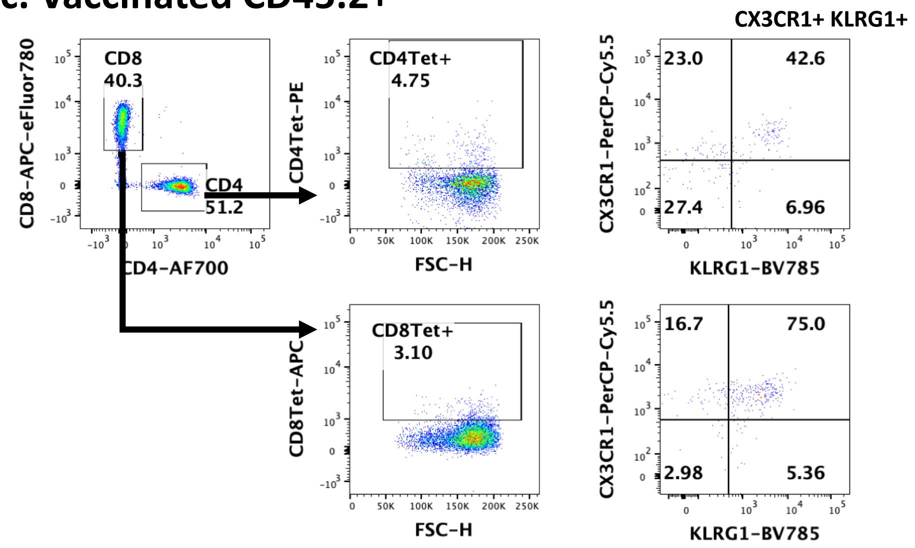

d. Naïve CD45.2+

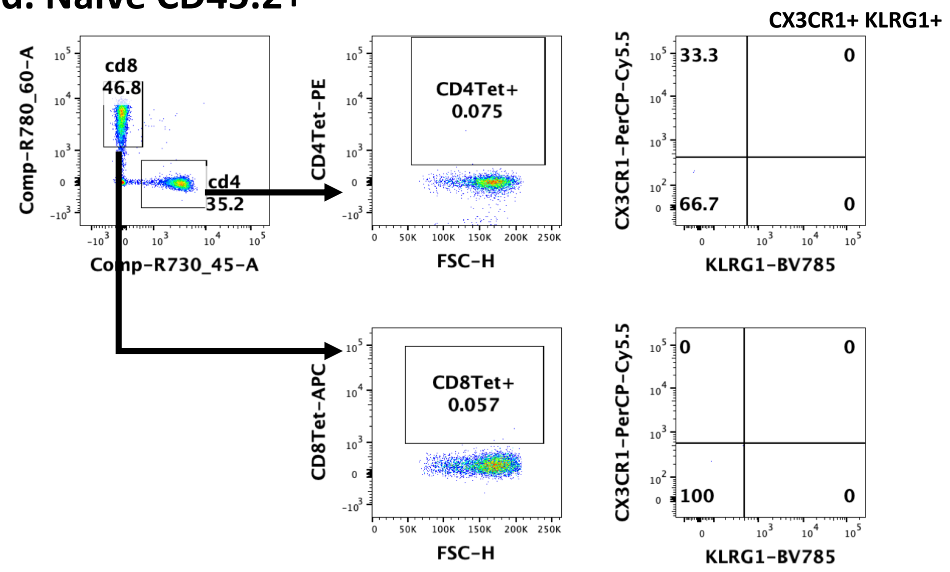

Supplementary Figure S2: Flow cytometry gating strategy for lung resident memory T cells. PPE15 I-A(b) and H-2D(b) tetramers were used in combination with intravascular staining with αCD45. Lymphocytes were gated based on forward scatter (FSC) and side scatter (SSC). After the exclusion of doublets, live CD45R- cells were selected. $CD3^{+}$ cells were subsequently gated into (a, b) CD45.2- (lung parenchymal) and (c, d) CD45.2+ (intravascular) populations. These populations were then gated for CD4+ and CD8+ T cells, followed by further gating for CD4+ PPE15 I-A(b) and CD8+ H-2D(b) tetramer-positive cells. CD45.2-, tetramer-positive cells were analysed for resident memory markers by gating on CXCR3+KLRG1- or CXCR3+PD1+ populations. CD45.2+, tetramer positive CD4+ and CD8+ T cells were gated on CX3CR1+KLRG1+. Representative plots shown from lung cells from vaccinated (a, c) and from (b, d) naïve animals.

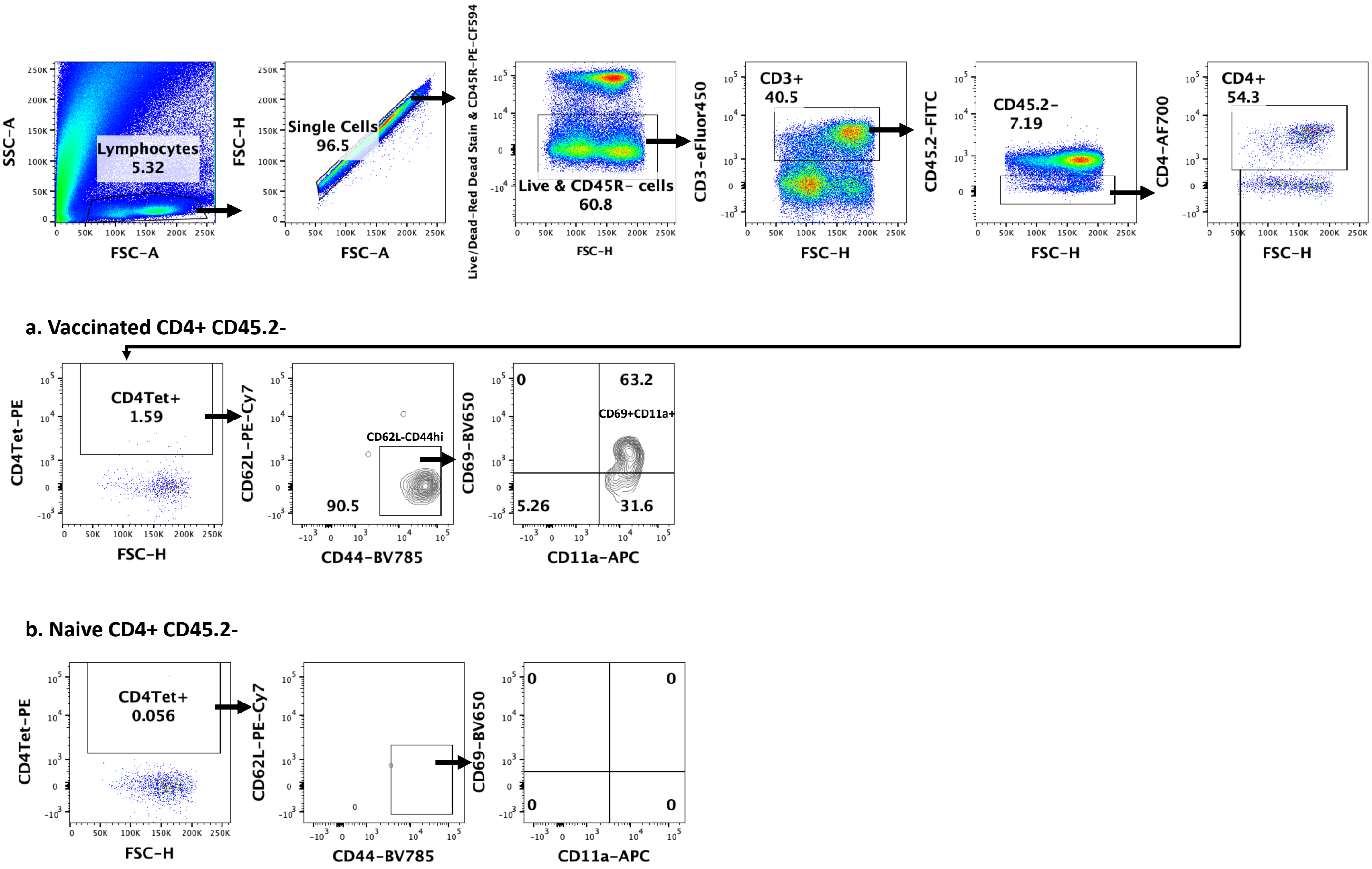

Supplementary Figure S3: Flow cytometry gating strategy for further characterisation of lung CD4+ resident memory T cells. Lymphocytes were gated based on forward scatter (FSC) and side scatter (SSC). After the exclusion of doublets, live CD45R- cells were selected. CD3+ cells were further gated into CD45.2- (lung parenchymal) populations. These populations were then gated for CD4+ T cells, followed by additional gating for CD4+ PPE15 I-A(b) tetramer-positive cells. Tetramer-positive cells were analysed for T effector memory markers by gating on CD62L-CD44hi populations, followed by further gating for CD69+CD11a+ cells to identify resident memory markers. Representative plots shown from cells derived from (a) vaccinated and (b) control animals.

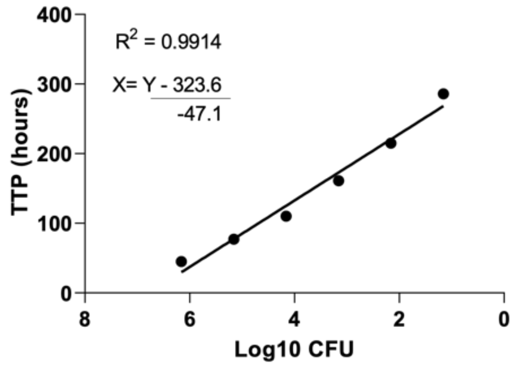

Supplementary Figure S4: BACTEC MGIT standard curve. Total CFU were calculated from the number of colonies counted from plating the bacterial stock. CFU were converted to log10 CFU, and linear regression analysis carried out by fitting a semi-log log line within GraphPad Prism. The R^2 value indicates the goodness of fit.

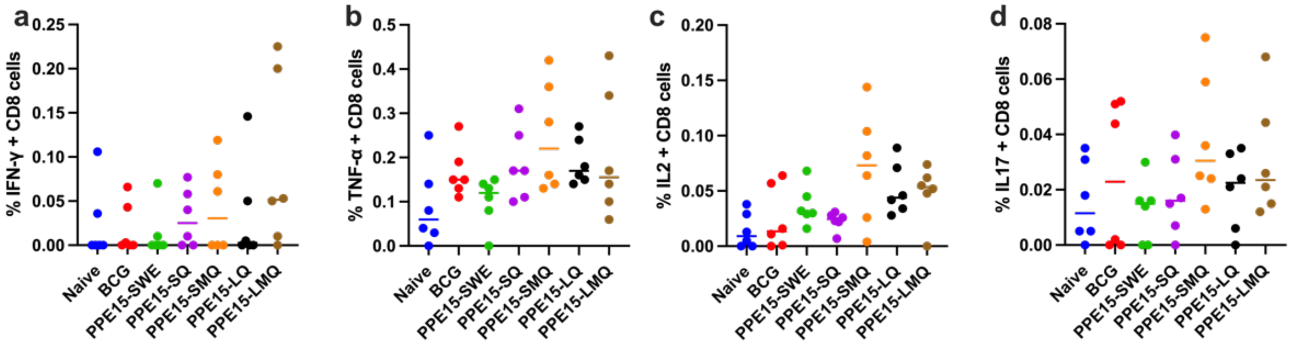

Supplementary Figure S5: CD8+ responses following vaccination with adjuvanted PPE15 protein. C57BL/6 mice were vaccinated twice with PPE15 protein formulated in five distinct adjuvants. Spleen cells from vaccinated and control animals were stimulated with PPE15 peptide pool to assess cytokine production. Proportion of CD8+ T cells expressing: (a) IFN-γ, (b) TNF-α, (c) IL-2, and (d) IL-17. Each symbol in the graphs represents an individual animal, and the lines the median response for each group. Statistical analyses were performed using Kruskal-Wallis, followed by Dunn's multi-comparison test to evaluate differences between groups.

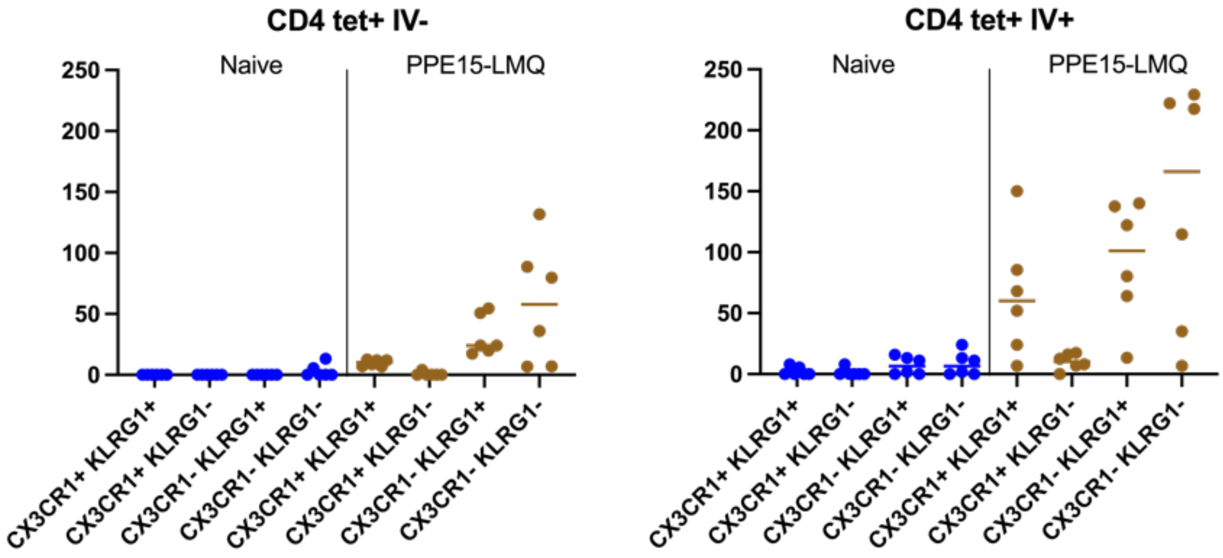

Supplementary Figure S6: Characterisation of lung immune responses in PPE15-LMQ vaccinated animals. Following vaccination with i.m. PPE15-LMQ, intravascular staining with αCD45 was conducted followed by tetramer and surface straining *in vitro*, to identify PPE15-specific cells. CX3CR1 and KLRG1 markers were then used on intravascular negative (IV-) and positive (IV+) populations.

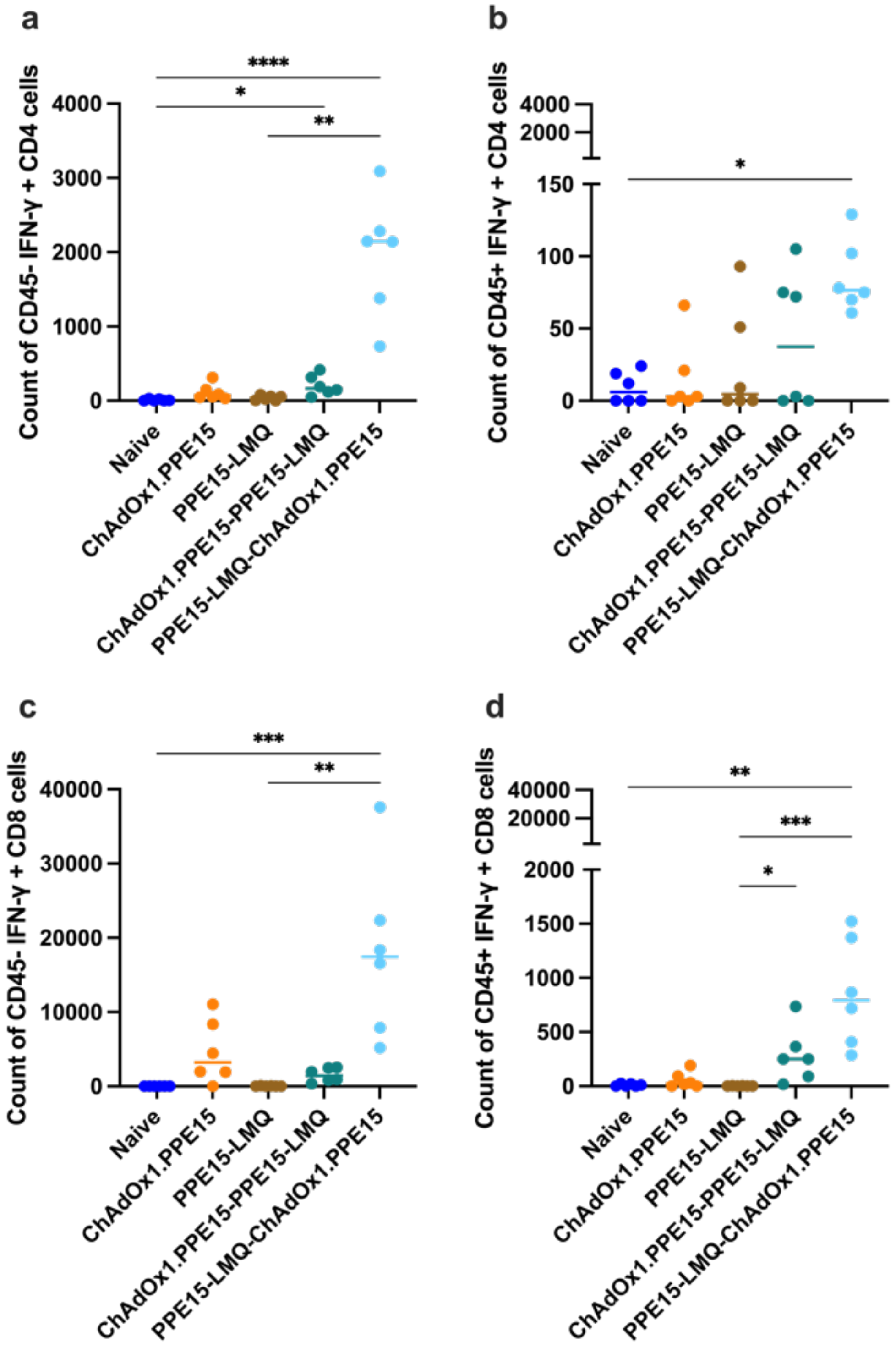

Supplementary Figure S7. Lung cellular responses following vaccination with heterologous regimes. Lung cells were harvested following intravascular staining with αCD45 and stimulated with PPE15 peptide pool. Number of CD4+ T cells expressing IFN-γ (a) intravascularly protected, (b) intravascularly stained. (c, d) Similar for CD8+ T cells. Each symbol in the graphs represents an individual animal, and the lines denote the median response for each group. Statistical analyses were performed using the Kruskal-Wallis test, followed by Dunn's multi-comparison test to evaluate differences between groups. Significance is denoted as follows: *p<0.05, **p<0.01, ***p<0.001.
